# Identification of Key Genes via Integrated Multi-Omics and Machine Learning Uncovers Tumor Biological Features and Prognostic Biomarkers in Uterine Leiomyosarcoma

**DOI:** 10.7150/ijms.126491

**Published:** 2026-02-04

**Authors:** Wei Lu, Susu Jiang, Qiran Sun, Yating Huang, Ying Yang, Xiaoqin Wang, Liwen Zhang, Yi Guo, Rujun Chen

**Affiliations:** 1 Department of Gynecology and Obstetrics, Shanghai East Hospital, Tongji University, Shanghai, China.; 2 Department of Gynecology and Obstetrics, Shanghai Fifth People's Hospital, Fudan University, Shanghai, China.; 3 Center of Community-Based Health Research, Fudan University, Shanghai, China.

**Keywords:** uterine leiomyosarcoma, machine learning, diagnostic model, tumor immune microenvironment, single-cell sequencing

## Abstract

**Background:**

Uterine leiomyosarcoma (ULMS) is a rare, aggressive uterine malignancy with high misdiagnosis rates, poor prognosis, and limited molecular biomarkers. Its pathogenesis, links between specific genes and the tumor immune microenvironment (TIME), and applications of machine learning (ML) and Mendelian randomization (MR) remain understudied.

**Methods:**

Multi-cohort data (4 GEO datasets, TCGA-SARC, single-cell sequencing) were integrated. Differentially expressed genes (DEGs) and WGCNA-derived key modules identified “InteGenes”. 113 ML algorithms were compared to build a diagnostic model (top: GBM, core genes = “Mgenes”). CIBERSORT analyzed TIME; MR explored Mgenes-ULMS causal links.

**Results:**

96 InteGenes enriched in cell cycle/p53/DNA repair pathways. The GBM model had training AUC = 1 and validation accuracy 92.3-100%; 36 Mgenes (e.g., TRIP13, AUC = 0.972) showed diagnostic value. Mgenes correlated with TIME (upregulated Mgenes ↔ M2 TAMs/Tregs; downregulated ↔ effector cells). MR found no genetic causality between Mgenes and ULMS.

**Conclusion:**

InteGenes reflect ULMS pathogenesis; the GBM model and Mgenes are promising diagnostic tools. Mgenes modulate ULMS's TIME, offering immunotherapeutic targets. This study advances ULMS molecular/immune understanding for translational research.

## 1. Introduction

Uterine leiomyosarcoma (ULMS) is a rare but highly aggressive malignant tumor of the uterine mesenchyme, accounting for approximately 1-2% of all uterine malignancies yet responsible for a disproportionate number of uterine cancer-related deaths^1^. Clinically, ULMS poses significant challenges: its nonspecific symptoms (e.g., abnormal uterine bleeding, pelvic pain) overlap with benign uterine lesions such as leiomyomas, leading to frequent misdiagnosis^1^. Moreover, ULMS exhibits strong invasiveness and early metastatic potential, with a 5-year overall survival rate of only 30-50% for advanced-stage disease^2^. Current diagnostic workflows for ULMS are dominated by postoperative histopathological assessment, a gold standard that is constrained by inter-observer variability and pathologist-dependent subjectivity. Notably, robust molecular biomarkers capable of predicting clinical outcomes, stratifying prognostic risk, and uncovering the biological underpinnings of tumor progression are still lacking, hindering the development of precision oncology approaches for this aggressive malignancy.

At the molecular level, ULMS pathogenesis is linked to dysregulation of core oncogenic pathways, including cell cycle progression, DNA repair, and tumor suppressor signaling (e.g., p53)^3^, ^4^. However, most previous studies have focused on single datasets or candidate genes, lacking systematic integration of multi-cohort transcriptomic data and rigorous validation—limiting the generalizability of their findings. Additionally, the tumor immune microenvironment (TIME) plays a pivotal role in ULMS progression, as immunosuppressive cell populations (e.g., M2-like tumor-associated macrophages [TAMs], regulatory T cells [Tregs]) promote immune escape^5^, ^6^; yet, the molecular links between ULMS-specific genes and TIME modulation remain poorly characterized. Furthermore, while machine learning (ML) has emerged as a powerful tool for developing diagnostic and prognostic models in oncology, its application to ULMS—particularly with large-scale algorithmic comparisons, multi-cohort validation, and a focus on linking genetic signatures to tumor biological behavior and clinical outcomes—has been rarely reported. Finally, whether ULMS-associated genes exert causal genetic effects on disease susceptibility (a key question for distinguishing driver vs. passenger genes) has not been addressed via Mendelian randomization (MR), a robust method to minimize confounding in observational studies.

**Figure 1:**
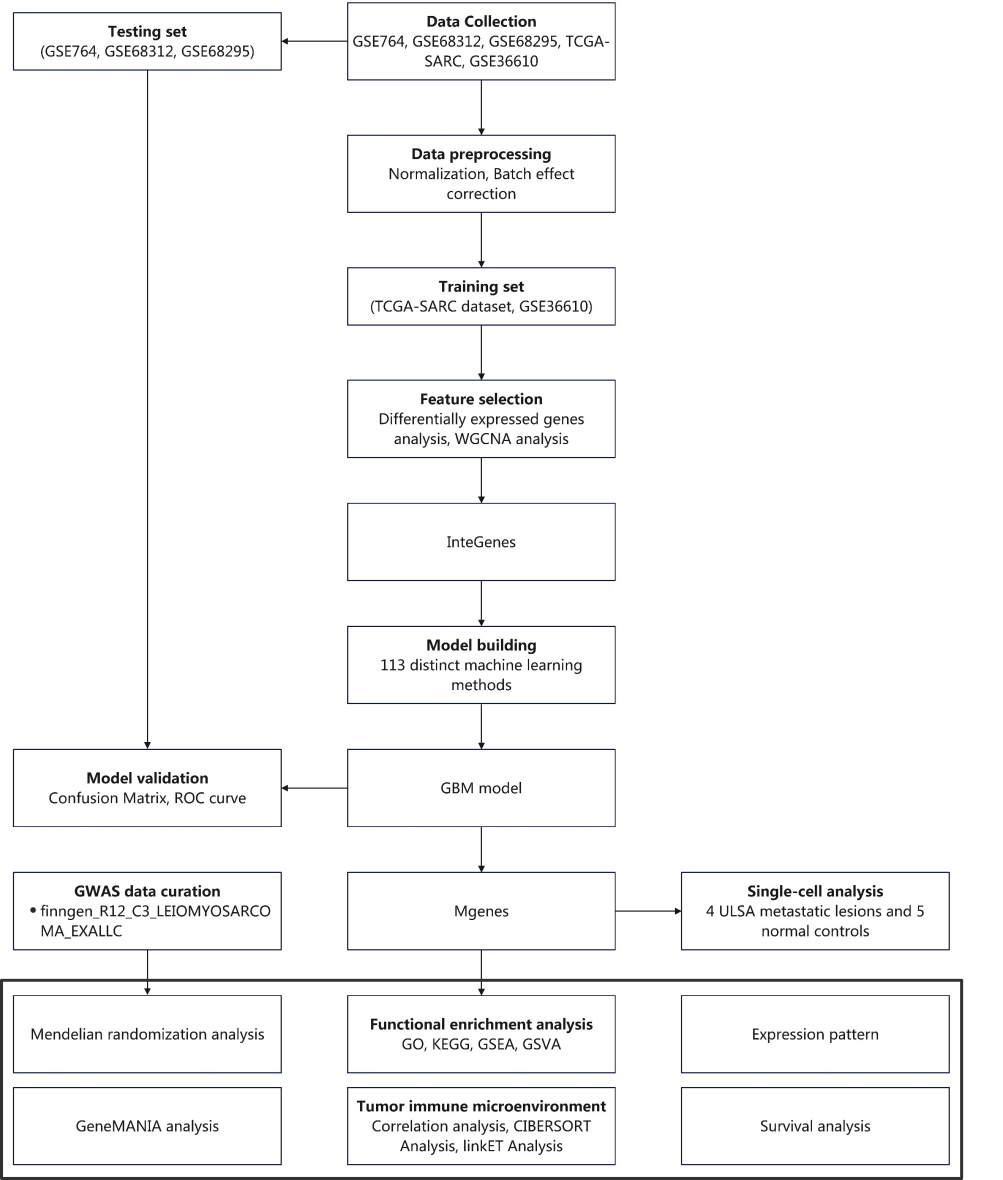
Research flow chart.

To address these gaps, the present study integrated multi-cohort transcriptomic data (4 GEO datasets + TCGA-SARC) and single-cell sequencing data to systematically investigate ULMS's molecular landscape. We first identified differentially expressed genes (DEGs) and key co-expression modules via weighted gene co-expression network analysis (WGCNA), then defined “InteGenes” as the intersection of DEGs and core module genes. Functional enrichment analyses (GO/KEGG) were performed to elucidate InteGenes' biological roles. We then compared 113 unique ML algorithms to construct and validate a high-performance diagnostic model, with the top model's core genes designated as “Mgenes.” Subsequently, we explored Mgenes' associations with TIME components via CIBERSORT and linkET analyses, and evaluated genetic causality between Mgenes and ULMS using MR. Collectively, this study aims to identify reliable diagnostic biomarkers, reveal Mgenes' roles in shaping the ULMS TIME, and provide a foundation for developing targeted and immunotherapeutic strategies. **Figure 1** presents an outline of the workflow.

## 2. Materials and Methods

### 2.1 Acquisition and preprocessing of datasets

In this study, 4 datasets of uterine leiomyosarcoma (ULMS) were incorporated from the Gene Expression Omnibus (GEO) database (https://www.ncbi.nlm.nih.gov/geo/), with each dataset containing sample data from both tumor groups and normal control groups. The detailed information of these datasets is as follows: GSE36610 (normal samples, N = 10; tumor samples, T = 12), GSE764 (normal samples, N = 4; tumor samples, T = 9), GSE68312 (normal samples, N = 3; tumor samples, T = 3), and GSE68295 (normal samples, N = 3; tumor samples, T = 6). Additionally, 27 cases of RNA-seq data and corresponding clinical data of ULMS were retrieved from the The Cancer Genome Atlas-Sarcoma (TCGA-SARC) dataset (https://portal.gdc.cancer.gov/) for inclusion in the study. Additionally, nine single-cell sequencing datasets were employed, specifically encompassing single-cell transcriptome sequencing data derived from 4 metastatic lesions of ULMS and 5 age-matched samples of normal myometrium, as described in previously published article^7^.

**Figure 2:**
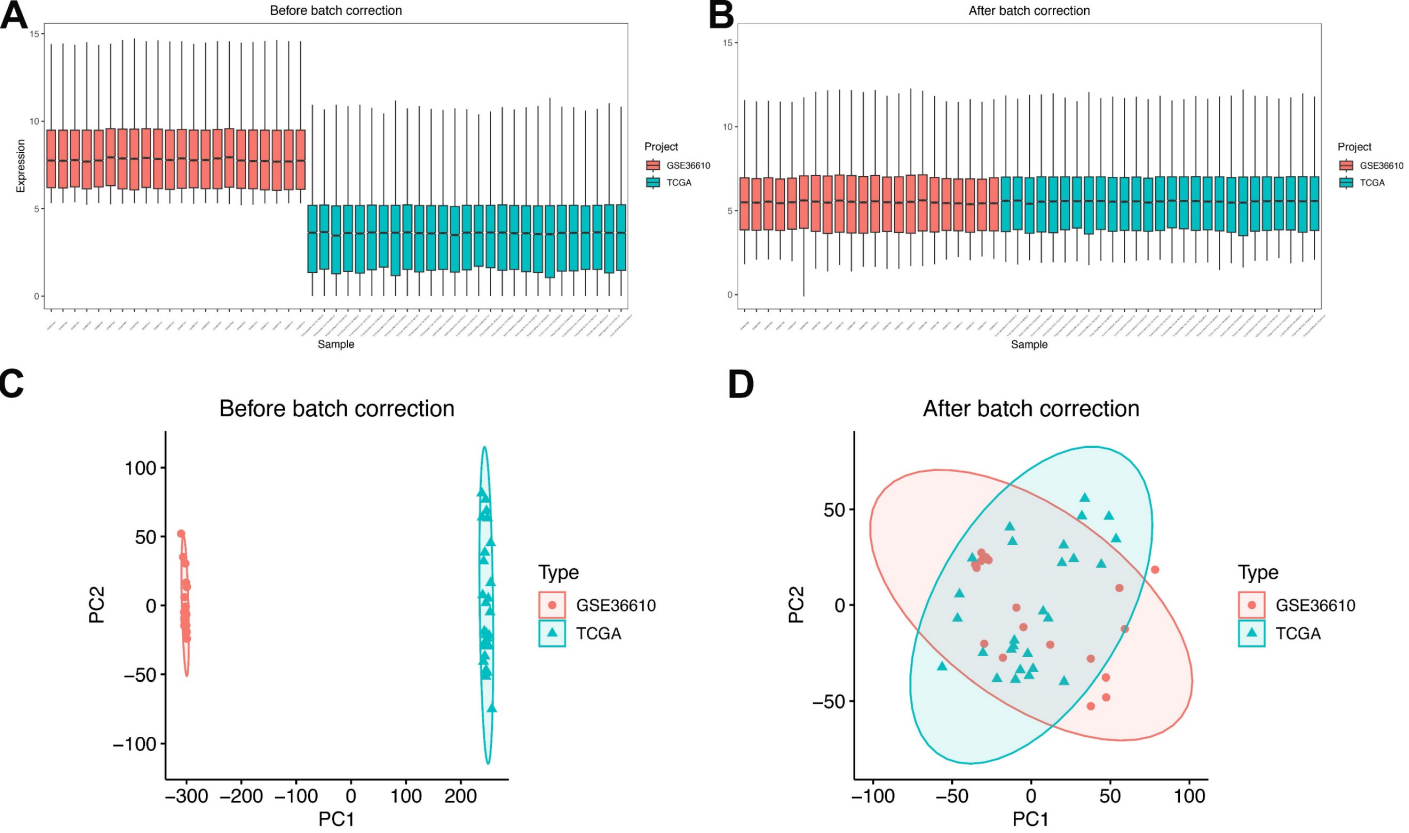
Data preparation. **(A)** Box plots showing expression profiles of each dataset before batch effect correction. **(B)** Box plots showing expression profiles of each dataset after batch effect correction.** (C)** PCA plots of each dataset before batch effect correction. **(D)** PCA plots of each dataset after batch effect correction.

For subsequent analytical procedures, the TCGA-SARC dataset and GSE36610 were designated as the training set, while GSE764, GSE68312, and GSE68295 were assigned as the validation set. Subsequently, the datasets within the training set were integrated, and batch effects—known to potentially introduce biases in gene expression data—were eliminated using the sva R package^8^. **Figure 2** presents the boxplots and principal component analysis (PCA) plots of the training set data both before and after batch effect removal. From these visualizations, it can be clearly observed that the batch effects were effectively mitigated, ensuring the reliability and comparability of the integrated training set data for subsequent downstream analyses.

### 2.2 Profiling of genes with differential

ExpressionGenes showing differential expression between tumor and normal samples in the training set were identified using the limma package^9^. The selection of these differentially expressed genes (DEGs) was based on two thresholds: a log fold change (logFC) of 1.5 and an adjusted P-value of 0.05. For visual representation of these DEGs, a heatmap was constructed utilizing the pheatmap package^10^, while a volcano plot was developed with the ggplot2 package^11^ to further illustrate the expression patterns.

### 2.3 Weighted gene co-expression network analysis

For weighted gene co-expression network analysis (WGCNA), we leveraged gene expression data from the integrated training set, which had undergone rigorous preprocessing and integration steps to guarantee data uniformity and quality. The WGCNA package^12^ was applied to conduct a thorough network analysis. Specifically, initial sample clustering was performed to assess inter-sample relationships and detect potential outliers, a step that helps uphold the robustness of subsequent network building. After sample clustering, module detection was carried out according to the co-expression patterns of genes; here, genes exhibiting comparable expression profiles across samples were clustered into separate modules. Finally, the gene inventory for each identified module was produced and exported, providing a basis for further functional enrichment analyses and investigations into module-trait associations.

### 2.4 Screening for intersection genes

The intersection of the previously identified DEGs and the genes from the key modules of WGCNA was calculated to obtain intersection genes. These genes were designated as InteGenes and used for subsequent research. Additionally, the VennDiagram package was utilized to generate a Venn diagram for visualizing this intersection.

### 2.5 Functional enrichment analysis

To explore the functional attributes of InteGenes, the R package “clusterProfiler”^13^ was employed to carry out comprehensive enrichment analyses encompassing Gene Ontology (GO) annotations and Kyoto Encyclopedia of Genes and Genomes (KEGG) pathway mapping. The GO analysis was designed to classify these genes into three primary categories—biological processes, cellular components, and molecular functions—thereby offering perspectives on their specific roles in diverse biological events. In parallel, the KEGG pathway analysis was performed to pinpoint signaling pathways with significant enrichment, aiding in clarifying how these InteGenes collectively engage in particular physiological mechanisms or pathological cascades. Together, these functional enrichment analyses establish a basis for unraveling the core biological functionalities and regulatory networks that underpin the phenotypic traits under investigation.

### 2.6 Construction of diagnostic models through 113 unique machine learning strategies

This study deployed a total of 113 distinct machine learning strategies for developing diagnostic models. Specifically, a comprehensive catalog of these models—encompassing their core features and foundational principles—is provided in **Supplementary Table 1** for thorough reference. Additionally, the algorithmic code implementing all 113 machine learning approaches, complete with respective parameter settings and computational pipelines, is available in **Supplementary Code 1**. Notably, among the variables integrated into the models, the smallest number of variables included in any constructed model was 5, ensuring a baseline complexity to capture meaningful diagnostic patterns. The model demonstrating the highest mean AUC in both the training and validation sets was selected and designated as "sModel" for subsequent analyses. ROC curves for the training and validation sets were generated using the pROC package^14^. Genes incorporated in this model were extracted and labeled as Mgenes, and volcano plots illustrating these Mgenes were subsequently created using the ggplot2 package.

### 2.7 Development of the confusion matrix

Since sModel was earlier established as the top-performing model—due to its superior mean AUC across both the training and validation sets—it was chosen to generate the confusion matrix. This process seeks to further gauge the model's classification abilities by specifying counts of true positives, true negatives, false positives, and false negatives, thereby offering a detailed overview of its predictive precision.

### 2.8 Correlation assessments

Using the integrated gene expression dataset, correlation assessments were conducted to explore expression associations among Mgenes, with the goal of uncovering potential co-expression patterns and interdependent relationships. To visually depict these correlative trends, comprehensive correlation diagrams were generated via the PerformanceAnalytics package^14^. These visuals effectively capture both the magnitude and direction of associations between each pair of Mgenes, providing clear insights into their interconnections.

### 2.9 GeneMANIA-based analyses

To explore in detail the functional connections and interaction profiles among Mgenes, we carried out GeneMANIA^15^ analyses through its specialized web-based platform (https://genemania.org/). This computational method synthesizes a range of biological datasets—including gene co-expression trends, protein-protein binding interactions, pathway enrichments, genetic interplay, and shared functional annotations—to build a holistic network. The analysis seeks to uncover potential functional cooperativity, regulatory links, and interconnected biological mechanisms among Mgenes, thus offering insights into their coordinated functions within the biological system being studied.

### 2.10 Gene set enrichment analysis (GSEA)

To comprehensively investigate the biological functionalities and pathway connections of Mgenes, we conducted Gene Set Enrichment Analysis (GSEA) via the clusterProfiler package—a robust R-based toolkit for functional enrichment investigations. For this analytical process, we employed the "c2.cp.kegg.Hs.symbols.gmt" reference gene set, a carefully compiled dataset from the Molecular Signatures Database (MSigDB). This particular gene set includes canonical pathways (cp) sourced from the Kyoto Encyclopedia of Genes and Genomes (KEGG), annotated with human (Hs) gene symbols, rendering it highly suitable for identifying enriched signaling and metabolic pathways among Mgenes. By utilizing this pathway-centered reference dataset, the GSEA not only measures the strength of enrichment for relevant biological pathways but also uncovers potential coordinated mechanisms that underlie the functional roles of Mgenes, thereby offering valuable insights into their collective participation in the biological system being examined.

### 2.11 Gene set variation analysis (GSVA)

To profile the pathway activity dynamics of Mgenes across samples and examine their functional significance, we conducted Gene Set Variation Analysis (GSVA) via the R-based GSVA package^16^. In contrast to conventional gene set enrichment approaches that center on predefined gene sets within a single cohort, GSVA converts gene-level expression data into gene set-level enrichment scores. This transformation enables quantitative evaluation of how pathways vary across multiple samples. For this analysis, we drew on the "c2.cp.kegg.Hs.symbols.gmt" reference gene set from the Molecular Signatures Database (MSigDB). This resource comprises curated canonical pathways (cp) originating from the Kyoto Encyclopedia of Genes and Genomes (KEGG), annotated with human (Hs) gene symbols. By harnessing this pathway-focused dataset, our GSVA sought to quantify the dynamic enrichment of KEGG pathways among Mgenes across samples. This effort aimed to uncover potential shifts in biological processes linked to Mgenes, shedding light on their context-specific functional roles within the system under study.

### 2.12 Single-cell analysis

Single-cell sequencing data of ULMS were analyzed using the Seurat package^17^, and the results of cell type identification are described in our previously published paper^7^. Gene expression feature plots were generated using the FeaturePlot function, and violin plots of gene expression were generated using the VlnPlot function.

### 2.13 Survival analysis

The clinical data and transcriptome data of TCGA-SARC were collated, and cases were divided into high-expression and low-expression groups based on the median expression value of the gene. Survival curves for the high- and low-expression groups were plotted using the survival package, with statistical significance and P-values annotated.

### 2.14 CIBERSORT-based analyses

To profile the immune cell makeup within the studied samples, we conducted CIBERSORT-based analyses^18^ on the integrated gene expression data. CIBERSORT is a commonly employed computational tool that calculates the relative abundances of 22 immune cell subtypes from bulk gene expression profiles by deconvolving mixed transcriptional signals. For this deconvolution process, the reference panel of immune cell transcriptional signatures—encompassing canonical expression profiles of distinct immune cell subsets—was adopted as detailed in **Supplementary Table 2**. This analysis aimed to quantify the prevalence of various immune cell populations (e.g., T cell subsets, B cells, macrophages, and neutrophils) across samples. In doing so, it sought to uncover potential links between the immune microenvironment and the biological scenario under investigation, as well as shed light on the cross-talk between Mgenes and immune modulation.

### 2.15 linkET analyses

To investigate putative connections between model genes (Mgenes) and immune cell profiles, we performed linkET analyses^19^, drawing on findings from our earlier CIBERSORT investigations. linkET is a computational method developed to unravel intricate interplay among biological attributes—here, its focus was on measuring and illustrating the cross-talk between Mgene expression patterns and the relative proportions of immune cell subsets as estimated by CIBERSORT. This analytical approach aimed to identify specific correlations (such as positive or negative associations) between individual Mgenes and distinct immune cell populations, while also pinpointing broader patterns of co-regulation. By integrating Mgene expression data with immune cell composition profiles, the linkET analyses sought to clarify how Mgenes might shape or be shaped by the immune microenvironment, thereby offering a more holistic understanding of their functional roles within the biological system being studied.

### 2.16 Correlation analysis of antineoplastic drug sensitivity

To investigate the correlation between the expression of Mgenes and sensitivity to antineoplastic drugs, we performed drug sensitivity analysis using the GSCA tool (https://guolab.wchscu.cn/GSCA/#/drug)^20^. This tool compiles the half-maximal inhibitory concentration (IC₅₀) values of 265 small-molecule compounds across 860 cell lines, together with the corresponding mRNA gene expression profiles, retrieved from the Genomics of Drug Sensitivity in Cancer (GDSC) database. The mRNA expression datasets and drug sensitivity datasets were integrated, followed by Pearson correlation analysis to determine the correlation between gene mRNA expression levels and drug IC₅₀ values. The P-values were adjusted using the false discovery rate (FDR) method. Additionally, this tool compiles the IC₅₀ values of 481 small-molecule compounds in 1001 cell lines and their corresponding mRNA gene expression profiles from the Genomics of Therapeutics Response Portal (CTRP). The mRNA expression datasets and drug sensitivity datasets were integrated in the same manner, and Pearson correlation analysis was conducted to assess the correlation between gene mRNA expression levels and drug IC₅₀ values, with P-values adjusted via the FDR method. For result visualization, the top 30 ranked drugs from the analyses were selected to generate a correlation heatmap.

### 2.17 GWAS data compilation and mendelian randomization (MR) analyses

To explore putative causal links between Mgenes and leiomyosarcoma pathogenesis, we first assembled relevant genomic datasets from two reputable repositories: the FinnGen database (https://www.finngen.fi/)^21^ and the IEU Open GWAS Project (https://opengwas.io/)^22^. Specifically, we obtained leiomyosarcoma-associated genome-wide association study (GWAS) datasets—capturing genetic variants tied to leiomyosarcoma susceptibility across large cohorts—and human gene expression quantitative trait locus (eQTL) datasets, which quantify relationships between genetic variants and Mgene expression levels. The GWAS dataset included in this study is detailed in **Table 1**.

Subsequent Mendelian Randomization (MR) analyses were conducted using the TwoSampleMR package in R, with Mgenes designated as exposure factors (i.e., their expression levels) and leiomyosarcoma incidence as the clinical outcome. To uphold methodological rigor, genetic instrumental variables (single nucleotide polymorphisms, SNPs) were selected from eQTL datasets based on strict criteria: they must reach genome-wide significance with Mgene expression (*P* < 5×10⁻⁸) to ensure robust associations; exhibit low linkage disequilibrium (r² < 0.001 within a 10,000 kb window) to avoid correlated instruments; and be excluded if located within 500 kb of known leiomyosarcoma susceptibility loci to reduce pleiotropy. The leiomyosarcoma GWAS dataset—used to quantify associations with the outcome (Y, leiomyosarcoma)—included cases and controls, ensuring sufficient power to detect genetic links to disease susceptibility. Meanwhile, the eQTL dataset—focused on capturing associations between instrumental variables (Z, SNPs) and the exposure (X, Mgene expression)—encompassed individuals, providing reliable estimates of SNP-Mgene expression correlations. Collectively, these datasets provided the statistical robustness necessary to detect modest causal effects in subsequent MR analyses, where Z acts as a genetic proxy for X to infer its causal relationship with Y.

Multiple MR analytical approaches were employed for cross-validation: inverse variance weighted (IVW) as the primary method to estimate overall causal effects using all valid IVs; MR-Egger regression to account for potential horizontal pleiotropy and deliver a pleiotropy-adjusted effect estimate; weighted median estimator, robust to up to 50% invalid IVs; and weighted mode estimator, which prioritizes IVs with consistent effect directions. Heterogeneity among IVs was evaluated using Cochran's Q statistic (significance threshold *P* < 0.05), with the I² statistic quantifying the proportion of variance due to heterogeneity. For pleiotropy testing, the MR-Egger intercept test (significance threshold *P* < 0.05) was applied to detect directional horizontal pleiotropy, and the MR-PRESSO (Mendelian Randomization Pleiotropy RESidual Sum and Outlier) method was used to identify and correct for outlier SNPs introducing pleiotropic bias. A leave-one-out sensitivity analysis was also performed to assess whether individual SNPs unduly influenced the overall effect estimate, ensuring result robustness. This comprehensive framework aimed to rigorously evaluate whether genetically predicted Mgene expression levels exert causal effects on leiomyosarcoma risk, leveraging two-sample MR to minimize confounding and reverse causation biases inherent in observational studies.

### 2.18 Statistical analyses

All statistical procedures carried out in the present study were implemented via R software (version 4.3.2)—a versatile platform extensively employed for statistical computation and data visualization in the field of biomedical research. To establish statistical significance, a strict threshold of p < 0.05 was employed, which ensures that any observed associations or discrepancies are not likely to stem from random variation. Furthermore, all graphical outputs—such as scatter plots, heatmaps, and volcano plots—were constructed using the ggplot2 package in R. This tool is a robust and adaptable component of the R ecosystem, enabling the development of high-fidelity, customizable visualizations that effectively showcase key insights derived from the analyses. This standardized methodology for statistical testing and visualization guarantees the reproducibility of study results and ensures clarity when presenting findings, aligning with rigorous biomedical research standards.

## 3. Results

### 3.1 Identification of differentially expressed genes based on transcriptome data from the training set

In the present study, a total of three distinct datasets were designated as the training cohort to support the analysis of transcriptome data, specifically including the TCGA-SARC dataset and the GSE36610 dataset. Together, these training datasets collectively contained 39 clinical samples derived from patients with uterine leiomyosarcoma, as well as 10 normal control samples that served as the baseline reference for comparison. Separately, three other datasets were assigned to serve as the testing cohort to validate subsequent findings, namely GSE764, GSE68312, and GSE68295. This testing cohort comprised 18 uterine leiomyosarcoma samples and an additional 10 normal control samples, ensuring consistency in the control group setup across both training and testing phases. To pinpoint genes with differential expression patterns between the uterine leiomyosarcoma group and the normal control group, the limma software package—widely used in bioinformatics for analyzing gene expression microarray and RNA-seq data—was employed for statistical analysis. This analytical process ultimately led to the identification of 143 differentially expressed genes (DEGs), which are critical for further exploring the molecular signatures of uterine leiomyosarcoma. Comprehensive details regarding the specific information of these 143 DEGs, such as their expression fold changes and statistical significance, are provided in **Supplementary Table 3** for reference.

### 3.2 Identification of key gene modules via weighted gene co-expression network analysis (WGCNA)

**Figure 3:**
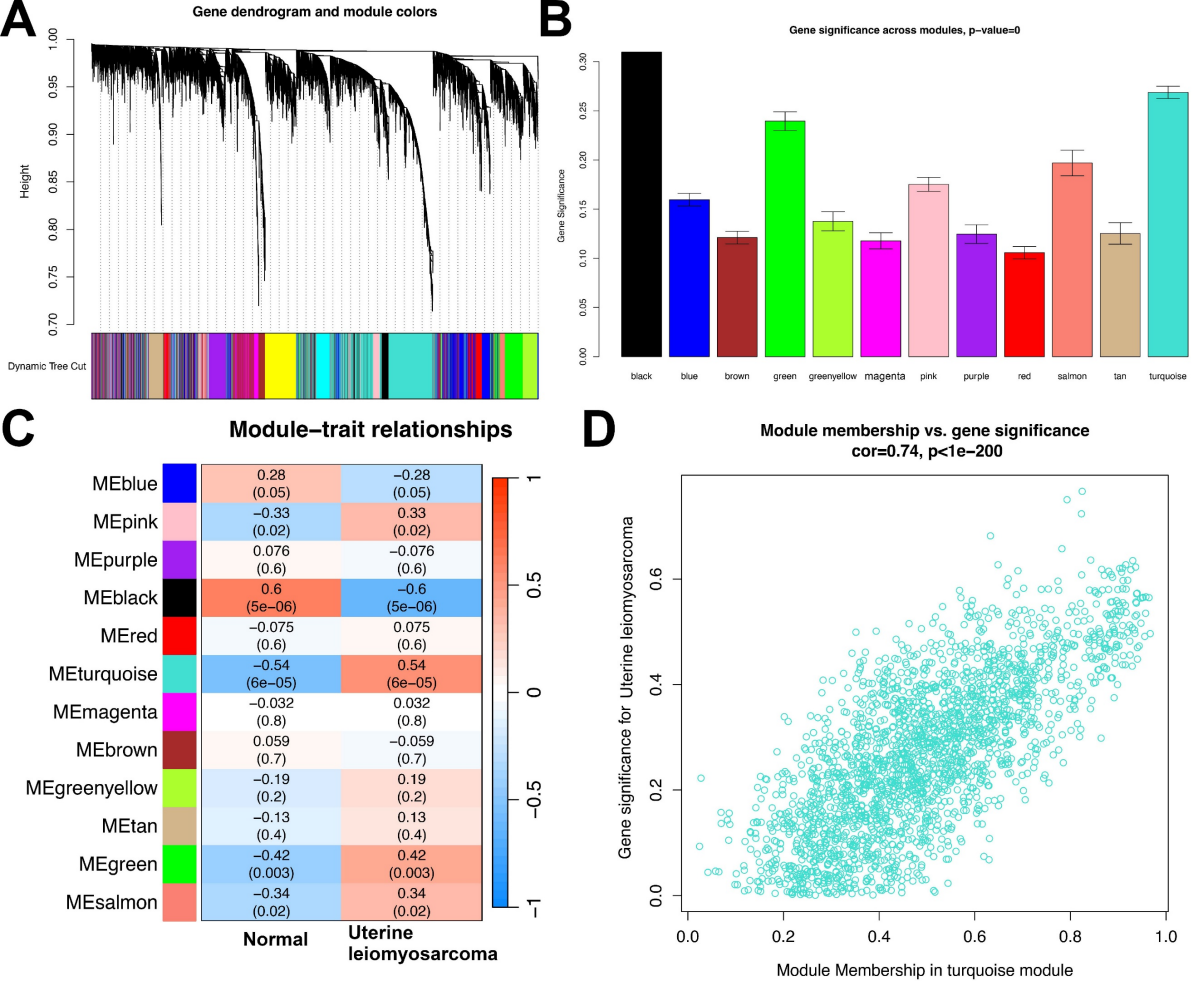
WGCNA Analysis. **(A)** Gene dendrogram with corresponding module colors. **(B)** Distribution of gene significance across different modules. **(C)** Module-trait relationship heatmap. Blue indicates a negative correlation, while red indicates a positive correlation.** (D)** Scatter plot of Module Membership (MEturquoise) vs. Gene Significance.

To pinpoint co-expression gene modules closely linked to uterine leiomyosarcoma, weighted gene co-expression network analysis (WGCNA) was conducted on the transcriptome data of the testing set. This analytical approach generated 12 distinct gene modules, each labeled with a unique color for straightforward differentiation and reference: MEblue, MEpink, MEpurple, MEblack, MEred, MEturquoise, MEmagenta, MEbrown, MEgreenyellow, MEtan, MEgreen, and MEsalmon (**Figures 3A and 3B**).

Among these 12 color-labeled modules, 5 were found to exhibit statistically significant associations with the uterine leiomyosarcoma phenotype. Specifically, 4 of these modules—MEpink, MEturquoise, MEgreen, and MEsalmon—showed strong positive correlations with the presence of uterine leiomyosarcoma. Their respective correlation coefficients were 0.33, 0.54, 0.42, and 0.34, with corresponding p-values of 0.02, 6×10⁻⁵, 0.003, and 0.02. This positive correlation pattern suggests that the coordinated expression trends of genes within these four modules may be upregulated during the development or progression of uterine leiomyosarcoma, potentially reflecting their role in driving disease-related molecular processes.

In contrast, one module—MEblack—displayed a notable significant negative correlation with the uterine leiomyosarcoma phenotype, with a correlation coefficient of -0.6 and a p-value of 5×10⁻⁶. This observation implies that the overall gene expression profile of the MEblack module is likely downregulated in uterine leiomyosarcoma tissues or samples, which may indicate a potential tumor-suppressive role of genes within this module (**Figure 3C**).

Given its robust positive correlation with the uterine leiomyosarcoma phenotype—particularly its relatively high correlation coefficient (0.54) compared to the other positively associated modules—MEturquoise was selected as the focal module for further in-depth analysis. **Figure 3D** presents a scatter plot that visualizes the relationship between two key metrics within MEturquoise: module membership (MM) and gene significance (GS). Module membership (MM) is a quantitative indicator that evaluates how strongly each individual gene is associated with the overall expression pattern of the MEturquoise module, thus reflecting the gene's centrality or “importance” within the module. Gene significance (GS), by contrast, quantifies the strength of the statistical link between each gene's expression level and the uterine leiomyosarcoma phenotype (e.g., whether the gene's expression differs significantly between tumor and normal samples).

The results from this scatter plot analysis revealed a strong and statistically robust positive correlation between GS and MM (correlation coefficient = 0.74, p < 1×10⁻²⁰⁰). This means that genes with high GS—i.e., those most strongly associated with the uterine leiomyosarcoma phenotype—also tend to have high MM, indicating they are among the most central and influential genes within the MEturquoise module. This finding not only validates the biological relevance of the MEturquoise module to uterine leiomyosarcoma pathogenesis but also highlights that the core genes of this module are tightly intertwined with the disease phenotype. Consequently, MEturquoise is reinforced as a critical module for subsequent investigations into the molecular mechanisms underlying uterine leiomyosarcoma.

### 3.3 Identification of intersection genes and functional enrichment analysis

**Figure 4:**
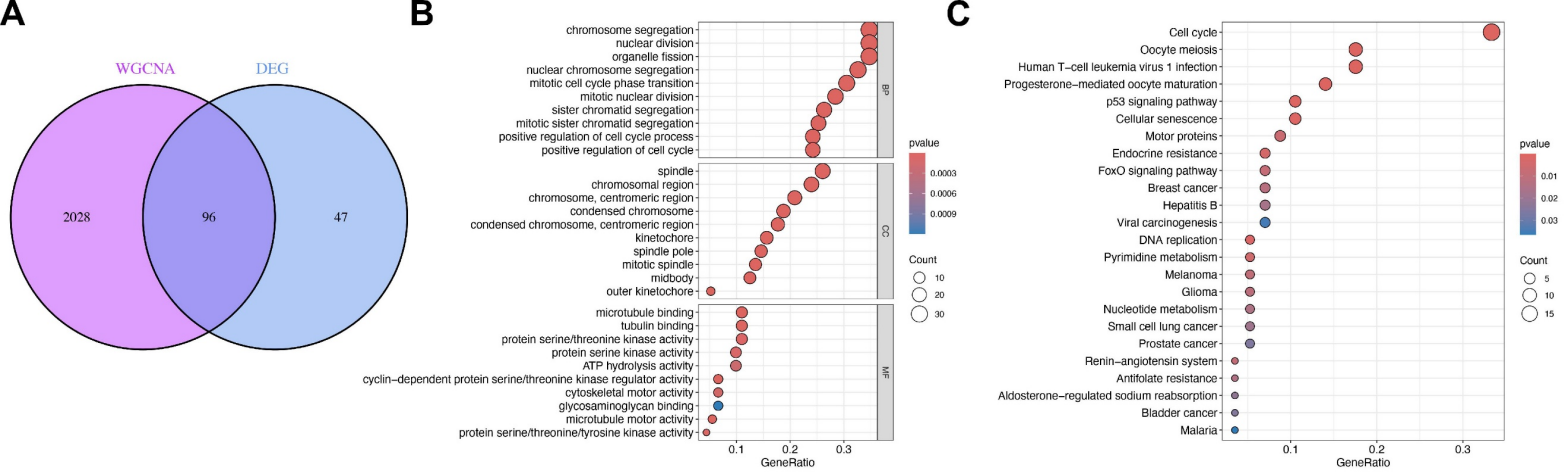
InteGenes functional enrichment analysis.** (A)** Venn diagram showing overlapping genes between WGCNA MEred module genes and DEGs. **(B)** GO Enrichment Analysis based on InteGenes. **(C)** KEGG Enrichment Analysis based on InteGenes.

To further narrow down and prioritize candidate genes that possess both differential expression profiles and a close association with the ULMS-relevant MEturquoise module, we determined the overlap between the previously identified differentially expressed genes (DEGs) and the 292 genes constituting the MEturquoise module—detailed information on these 292 module genes is available in Supplementary Table 4. This intersection analysis yielded 96 shared genes, which were formally designated as “InteGenes” (a portmanteau of “Intersection Genes”) to reflect their dual origin from both DEGs and the MEturquoise module (**Figure 4A**). These InteGenes represent a highly prioritized candidate set, as they simultaneously meet two critical criteria: they exhibit significant expression differences between ULMS tumor samples and normal control samples, and they play central roles in the MEturquoise module—a module already linked to ULMS pathogenesis. This dual qualification not only filters out genes with potential non-specific relevance but also enhances their credibility as functionally important targets for investigating the molecular mechanisms underlying ULMS.

To delineate the biological functions and molecular roles of these InteGenes, we subsequently performed two complementary enrichment analyses: Gene Ontology (GO) enrichment analysis and Kyoto Encyclopedia of Genes and Genomes (KEGG) pathway enrichment analysis. For the GO analysis, results were systematically categorized into three core domains—Biological Process (BP), Cellular Component (CC), and Molecular Function (MF)—to provide a multi-dimensional view of InteGene-related biological activities. In the Biological Process (BP) domain, the top three most significantly enriched terms were chromosome segregation, nuclear division, and organelle fission; these processes are all tightly linked to cell division, where chromosome segregation ensures accurate distribution of genetic material during mitosis, and nuclear division and organelle fission serve as foundational steps in cell cycle progression—pathways frequently dysregulated in cancer to drive uncontrolled tumor cell proliferation, a key hallmark of ULMS. In the Cellular Component (CC) domain, the top three enriched terms included “spindle,” “chromosomal region,” and “chromosome, centromeric region”; the spindle is a microtubule-based structure essential for proper chromosome alignment and separation during cell division, while the chromosomal region (especially the centromeric region) is critical for maintaining chromosomal stability—a feature often compromised in ULMS, contributing to tumor heterogeneity and aggressiveness. In the Molecular Function (MF) domain, the top three enriched terms were microtubule binding, tubulin binding, and protein serine/threonine kinase activity; microtubule and tubulin binding are pivotal for cytoskeletal dynamics, which regulate cell shape, division, and migration, while protein serine/threonine kinase activity mediates intracellular signaling cascades that control cell growth and survival—both of which are core processes disrupted in ULMS tumorigenesis (**Figure 4B**).

Parallel to the GO analysis, KEGG pathway enrichment analysis was conducted to uncover the signaling and metabolic pathways in which InteGenes are actively involved, providing insights into their contextual roles in ULMS biology. This analysis identified 10 primary pathways with significant enrichment, each bearing relevance to cancer development or ULMS-specific molecular features: Cell cycle, a pathway frequently dysregulated in ULMS as aberrant cell cycle control drives uncontrolled tumor growth; Oocyte meiosis and Progesterone-mediated oocyte maturation, both linked to hormone-dependent cellular processes that align with ULMS's known responsiveness to steroid hormones; Human T-cell leukemia virus 1 (HTLV-1) infection, implicating potential virus-associated pro-tumorigenic mechanisms that may interact with ULMS pathogenesis; p53 signaling pathway, a well-characterized tumor suppressor pathway that regulates cell cycle arrest and apoptosis—dysregulation of this pathway is common in aggressive sarcomas like ULMS, contributing to treatment resistance; Cellular senescence, a process that restricts abnormal cell proliferation and is often inactivated in tumors to sustain malignant growth; Motor proteins, critical for cytoskeletal movement and cell division, supporting the GO findings on microtubule function and reinforcing the role of cell division dysregulation in ULMS; Endocrine resistance, relevant to ULMS clinical management as endocrine therapies are sometimes used in advanced cases, and resistance to such treatments remains a key challenge; FoxO signaling pathway, which regulates cell metabolism and stress responses, with established links to cancer progression and metastasis; and Breast cancer, highlighting potential shared molecular mechanisms between ULMS and other hormone-driven malignancies, offering opportunities for cross-disease translational research (**Figure 4C**).

Collectively, these GO and KEGG enrichment results paint a comprehensive picture of the biological roles of InteGenes, directly linking them to core cellular processes (e.g., cell division, cytoskeletal regulation) and signaling pathways (e.g., cell cycle, p53) that are known to be dysregulated in ULMS development and progression. By bridging differential gene expression with module-specific relevance and functional context, these findings not only validate the importance of InteGenes as key candidates for further study but also lay a foundation for subsequent functional experiments to confirm their role as mediators of ULMS pathogenesis—ultimately providing potential targets for the development of more effective diagnostic markers or therapeutic strategies for ULMS.

### 3.4 Construction and validation of machine learning models

**Figure 5:**
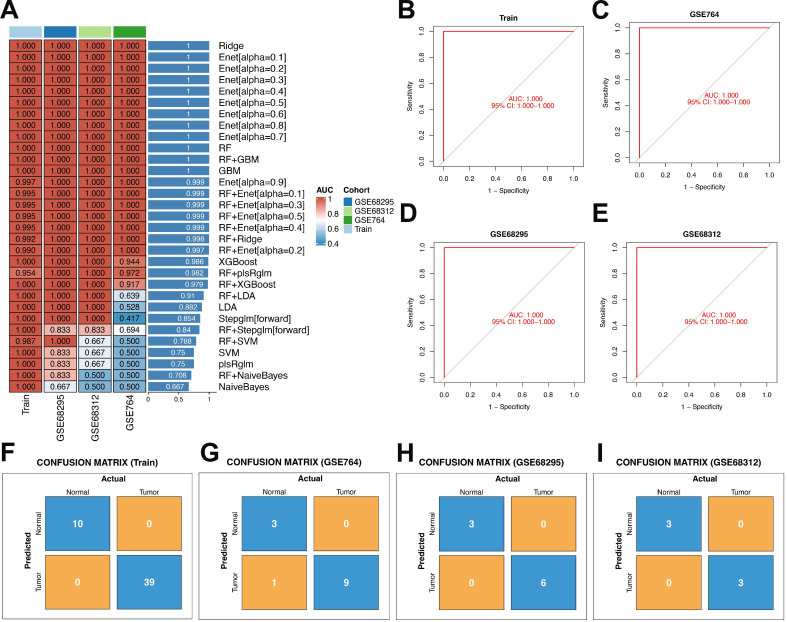
Construction and validation of diagnostic models via multiple machine learning methods. **(A)** Heatmap of AUC values for machine learning methods in the training set and test set. **(B)** ROC curve for the training set based on the GBM model.** (C)** ROC curve for GSE764 based on the GBM model. **(D)** ROC curve for GSE68295 based on the GBM model. **(E)** ROC curve for GSE68312 based on the GBM model. **(F)** Confusion matrix plot for the training set. **(G)** Confusion matrix plot for GSE764. **(H)** Confusion matrix plot for GSE68295.** (H)** Confusion matrix plot for GSE68312.

A total of 113 unique machine learning algorithms were utilized to develop diagnostic models for uterine leiomyosarcoma, and the gene parameters associated with each model are elaborated in Supplementary Table 5. Subsequently, these models underwent validation in three independent datasets (GSE764, GSE68312, and GSE68295), and the area under the curve (AUC) was calculated for each model, as depicted in **Figure 5A**. Upon sorting the models by AUC values in descending order, the top 12 performers were determined to be Ridge, Enet[α = 0.1], Enet[α = 0.2], Enet[α = 0.3], Enet[α = 0.4], Enet[α = 0.5], Enet[α = 0.6], Enet[α = 0.7], Enet[α = 0.8], Random Forest (RF), RF+Gradient Boosting Machine (GBM), and GBM. The average AUC of these top 12 models—derived from the mean of their AUC scores in the training set—was uniformly 1.

For a more in-depth assessment of model performance, the top 12 models (those with the highest average AUC) were subjected to confusion matrix analysis. Following this thorough evaluation, the GBM model was selected for subsequent investigations, as it demonstrated an AUC of 1 in the training set (**Figures 5B-E**). Evaluating the GBM model's performance through confusion matrices provided detailed insights: the confusion matrix for the training set revealed flawless classification between Normal (negative class) and Tumor (positive class). Specifically, all 10 actual Normal samples were correctly classified as Normal (true negatives, with a count of 10), and all 39 actual Tumor samples were accurately designated as Tumor (true positives, with a count of 39), with no false positives (counting 0, meaning no Normal samples were incorrectly labeled as Tumor) and no false negatives (counting 0, indicating no Tumor samples were misclassified as Normal). As a result, crucial metrics such as overall accuracy, Tumor-class precision, Tumor-class recall, and Normal-class specificity all attained 100%. Although the model successfully learned the discriminative features between classes in the training set, its ability to generalize needed validation using independent test sets to verify real-world applicability (**Figure 5F**). Subsequently, confusion matrix analysis was conducted on the three validation datasets.

Across the three independent validation datasets (GSE764, GSE68295, and GSE68312), the model displayed consistent and robust classification capability. In the GSE764 dataset, the overall accuracy reached approximately 92.3% (calculated as (true negatives + true positives) divided by total samples, which equals (3 + 9) divided by (3 + 1 + 9 + 0)). The precision for the Tumor class (the ratio of truly Tumor samples among all predicted Tumor samples) was 90% (calculated as true positives divided by (true positives + false positives), which equals 9 divided by (9 + 1)), the recall for the Tumor class (sensitivity, the proportion of truly Tumor samples correctly detected) was 100% (calculated as true positives divided by (true positives + false negatives), which equals 9 divided by (9 + 0)), and the specificity for the Normal class (the proportion of truly Normal samples correctly identified) was 75% (calculated as true negatives divided by (true negatives + false positives), which equals 3 divided by (3 + 1)) (**Figure 5G**). For the GSE68295 dataset, the overall accuracy was 100% (calculated as (true negatives + true positives) divided by total samples, which equals (3 + 6) divided by (3 + 0 + 6 + 0)). The Tumor-class precision, Tumor-class recall, and Normal-class specificity all reached 100% (calculated as 6 divided by (6 + 0), 6 divided by (6 + 0), and 3 divided by (3 + 0), respectively) (**Figure 5H**). In the GSE68312 dataset, the overall accuracy also reached 100% (calculated as (true negatives + true positives) divided by total samples, which equals (3 + 3) divided by (3 + 0 + 3 + 0)), and the Tumor-class precision, Tumor-class recall, and Normal-class specificity were all 100% (calculated as 3 divided by (3 + 0), 3 divided by (3 + 0), and 3 divided by (3 + 0), respectively) (**Figure 5I**).

Taken together, these metrics illustrate the model's robust generalizability: the perfect Tumor recall (100%) across all datasets ensures that all actual Tumor samples are detected, while the high-to-perfect accuracy, precision, and Normal specificity underscore the model's reliability in differentiating between Normal and Tumor classes—thus supporting its potential for practical use in clinical settings.

### 3.5 Expression patterns of Mgenes in uterine leiomyosarcoma

**Figure 6:**
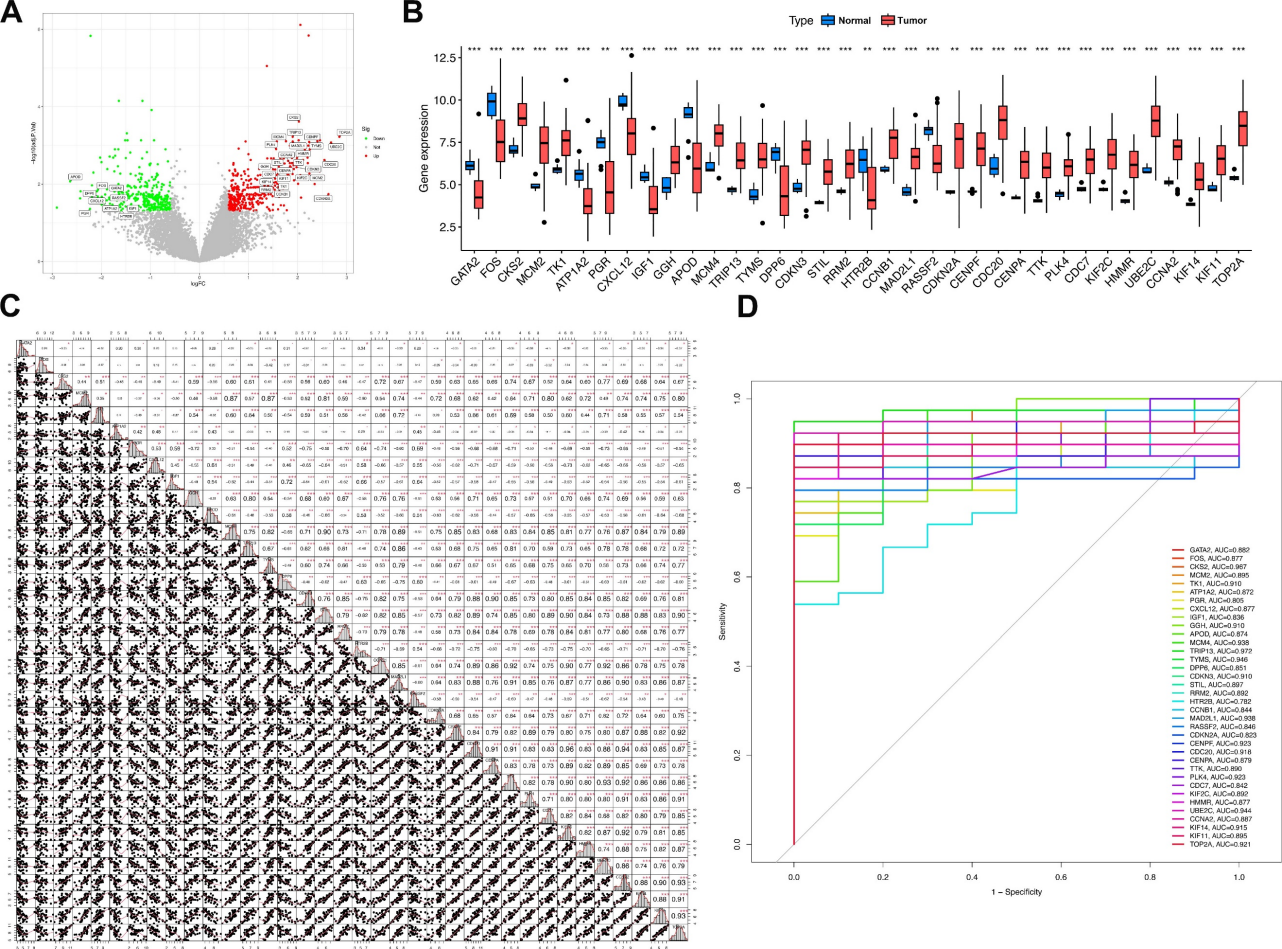
Expression patterns and expression correlation analysis of Mgenes. **(A)** Volcano plot of differential expression for Mgenes. **(B)** Expression differences of Mgenes between the tumor group and normal group. **(C)** Expression correlation plot among Mgenes. **(D)** ROC curves for single Mgenes. *P < 0.05, **P < 0.01, ***P < 0.001.

Thirty-six genes—specifically GATA2, FOS, CKS2, MCM2, TK1, ATP1A2, PGR, CXCL12, IGF1, GGH, APOD, MCM4, TRIP13, TYMS, DPP6, CDKN3, STIL, RRM2, HTR2B, CCNB1, MAD2L1, RASSF2, CDKN2A, CENPF, CDC20, CENPA, TTK, PLK4, CDC7, KIF2C, HMMR, UBE2C, CCNA2, KIF14, KIF11, and TOP2A—were integrated into the GBM model for subsequent analytical procedures. Among these 36 candidate genes, 10 (APOD, PGR, DPP6, CXCL12, FOS, ATP1A2, IGF1, GATA2, RASSF2, and HTR2B) displayed a statistically significant downregulated expression profile in uterine leiomyosarcoma tissues relative to non-tumor control tissues. In contrast, the remaining 26 genes—including CKS2, MCM2, TK1, GGH, MCM4, TRIP13, TYMS, CDKN3, STIL, RRM2, CCNB1, MAD2L1, CDKN2A, CENPF, CDC20, CENPA, TTK, PLK4, CDC7, KIF2C, HMMR, UBE2C, CCNA2, KIF14, KIF11, and TOP2A—exhibited a notable upregulation in expression levels within the tumor group (as shown in **Figure 6A, B**). Additionally, we also investigated the expression abundances of Mgenes across different cell types in the single-cell RNA sequencing data. Except for FOS, most Mgenes exhibited relatively low expression abundances (**Supplementary Figures 1 and 2**, where red indicates the normal group and green indicates the tumor group in the violin plots).

To further investigate the potential regulatory interactions and co-expression features of these Mgenes, a correlation analysis was performed on their expression levels. Results of this analysis uncovered distinct, biologically meaningful correlations between the expression profiles of the Mgenes, indicating the presence of potential synergistic or antagonistic regulatory networks among these genes during the progression of uterine leiomyosarcoma (**Figure 6C**).

Subsequently, to assess the diagnostic utility of each individual Mgene for differentiating uterine leiomyosarcoma tissues from non-tumor tissues, receiver operating characteristic (ROC) curves were constructed for each gene, and the associated area under the curve (AUC) values—indicators of diagnostic accuracy—were computed (**Figure 6D**). These AUC values were as follows: GATA2 (0.882), FOS (0.877), CKS2 (0.967), MCM2 (0.895), TK1 (0.910), ATP1A2 (0.872), PGR (0.805), CXCL12 (0.877), IGF1 (0.836), GGH (0.910), APOD (0.874), MCM4 (0.938), TRIP13 (0.972), TYMS (0.946), DPP6 (0.851), CDKN3 (0.910), STIL (0.897), RRM2 (0.892), HTR2B (0.782), CCNB1 (0.844), MAD2L1 (0.938), RASSF2 (0.846), CDKN2A (0.823), CENPF (0.923), CDC20 (0.918), CENPA (0.879), TTK (0.890), PLK4 (0.923), CDC7 (0.842), KIF2C (0.892), HMMR (0.877), UBE2C (0.944), CCNA2 (0.887), KIF14 (0.915), KIF11 (0.895), and TOP2A (0.921). Notably, TRIP13 exhibited the highest diagnostic performance, with an AUC of 0.972, whereas HTR2B had the relatively lowest—yet still clinically relevant—diagnostic accuracy (AUC = 0.782) among the 36 Mgenes. Collectively, these results suggest that most of the Mgenes possess substantial potential as valuable diagnostic biomarkers for uterine leiomyosarcoma.

### 3.6 Functional enrichment analysis of Mgenes

**Figure 7:**
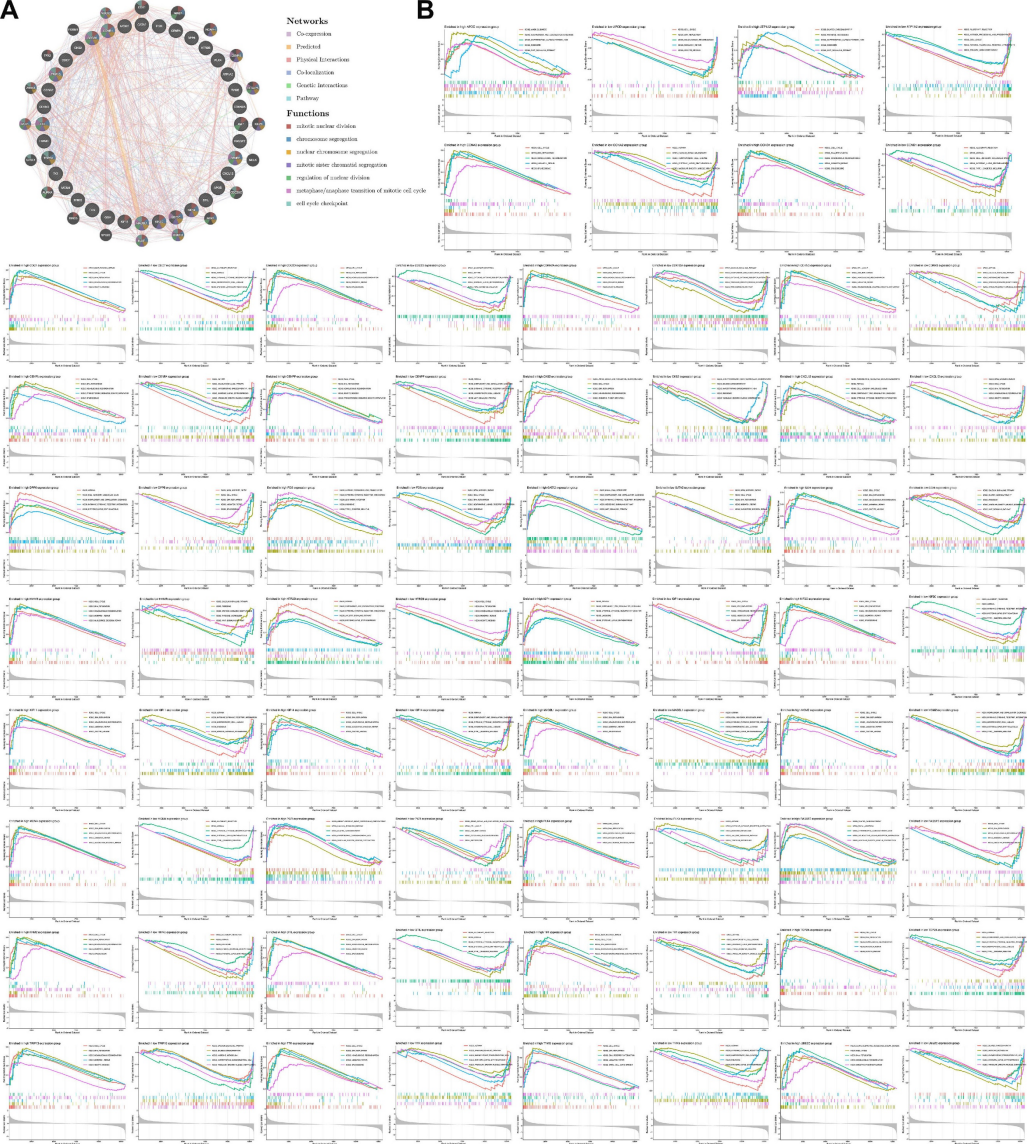
Protein-protein interaction network and GSEA analysis of Mgenes. **(A)** Protein-protein interaction network diagram of Mgenes. **(B)** GSEA plot for Mgenes.

To explore the underlying molecular interaction networks and functional implications of Mgenes in uterine leiomyosarcoma, we initially carried out protein-protein interaction (PPI) network analysis using the GeneMANIA bioinformatics platform (**Figure 7A**). This analysis identified 20 genes that display significant co-expression correlations with the 36 Mgenes, including NDC80, CDK1, MKI67, NCAPH, CENPE, DLGAP5, KIF23, MELK, CDC25C, NEK2, BUB1B, BUB1, SPC25, BIRC5, AURKA, GTSE1, PLK1, ZWINT, TPX2, and FOXM1. Notably, these co-expressed genes are well-documented to mediate core biological processes such as mitotic nuclear division, chromosome segregation, nuclear chromosome segregation, mitotic sister chromatid segregation, regulation of nuclear division, the metaphase-to-anaphase transition of the mitotic cell cycle, and cell cycle checkpoint control. Dysregulation of these processes is a hallmark of malignant transformation, as it drives uncontrolled cell proliferation, genomic instability, and aberrant cell cycle progression—all of which are key pathological features contributing to the initiation, invasion, and metastasis of uterine leiomyosarcoma. This suggests that Mgenes may interact with these co-expressed genes to modulate critical oncogenic pathways in the disease.

Subsequently, to gain deeper insights into the pathway enrichment profiles specific to each individual Mgene, we performed separate Gene Set Enrichment Analysis (GSEA) for all 36 Mgenes. The results of this gene-specific GSEA are illustrated in **Figure 7B** (with each subpanel corresponding to one Mgene), and consistently pointed to the enrichment of several key Kyoto Encyclopedia of Genes and Genomes (KEGG) pathways. These pathways included KEGG_CELL_CYCLE (a central regulator of cell proliferation, whose dysregulation is ubiquitous in sarcomas), KEGG_DNA_REPLICATION (essential for maintaining proper DNA duplication during cell division), and three DNA repair-related pathways—KEGG_BASE_EXCISION_REPAIR, KEGG_HOMOLOGOUS_RECOMBINATION, and KEGG_MISMATCH_REPAIR. Impairments in these DNA repair pathways are particularly relevant to uterine leiomyosarcoma, as they can lead to the accumulation of genetic mutations and chromosomal abnormalities, further promoting tumor progression and resistance to therapeutic interventions.

**Figure 8:**
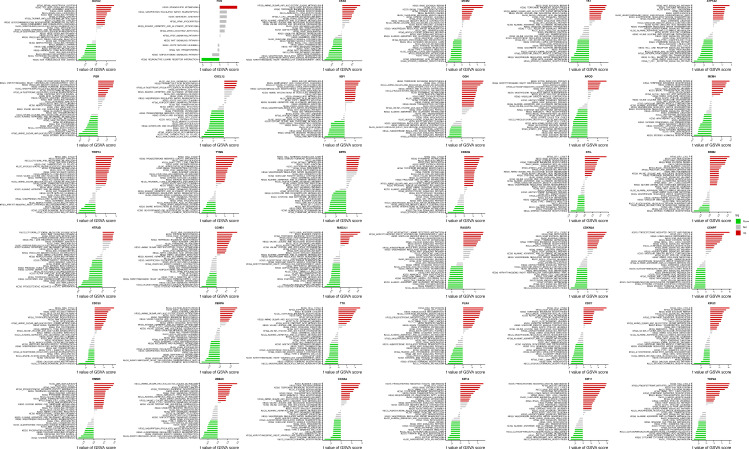
GSVA analysis based on Mgenes.

To validate the robustness and reproducibility of these enrichment findings, we additionally performed Gene Set Variation Analysis (GSVA) on the entire Mgene set. As shown in **Figure 8**, the GSVA results exhibited a high degree of concordance with the GSEA outcomes: both analyses consistently highlighted the enrichment of the aforementioned cell cycle, DNA replication, and DNA repair pathways. This cross-validation not only confirms the reliability of our functional enrichment results but also underscores that the association of Mgenes with these cancer-relevant pathways is not an artifact of individual gene analysis, but rather a collective functional characteristic of the entire Mgene set. Together, these findings strongly support the notion that Mgenes play a coordinated role in regulating critical oncogenic processes in uterine leiomyosarcoma, providing a molecular basis for their potential as diagnostic biomarkers and therapeutic targets.

### 3.7 Analysis of the impact of Mgenes expression on the survival prognosis of ULMS

**Figure 9:**
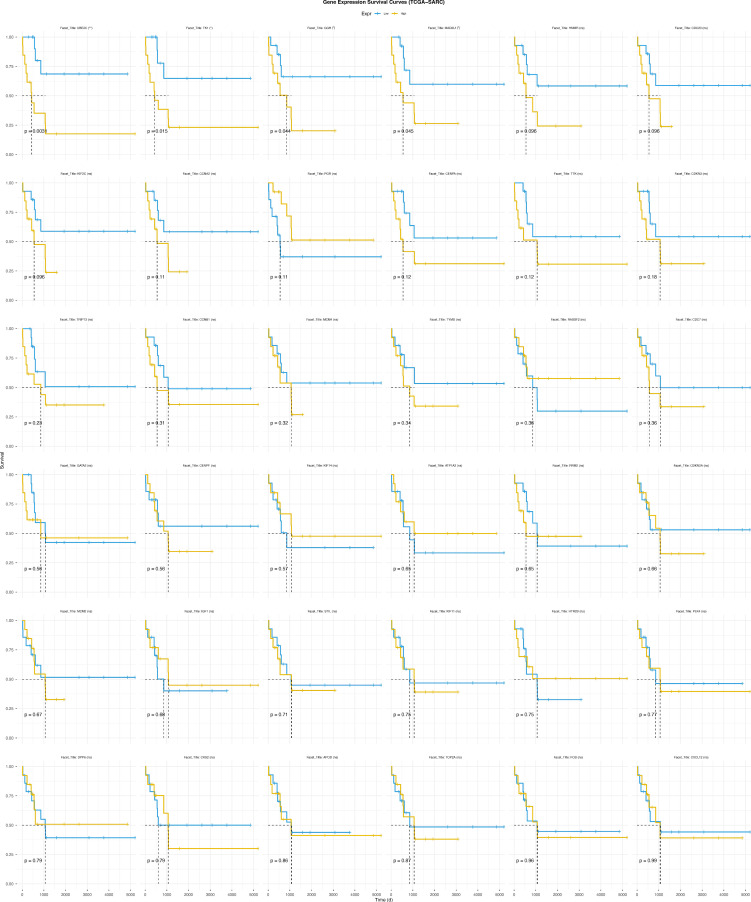
Survival curves of survival analysis on TCGA-SARC dataset grouped by Mgenes expression.

To investigate the impact of Mgenes expression on the survival prognosis of ULMS, we performed survival analysis using clinical data and transcriptomic data from the TCGA-SARC dataset. We found that UBE2C, TK1, GGH, and MAD2L1 (among Mgenes) were significantly associated with the survival prognosis of ULMS, and high expression of these genes was correlated with poor survival outcomes in ULMS (**Figure 9**).

### 3.8 Correlation analysis of Mgenes with diverse immune cells in the tumor immune microenvironment

**Figure 10:**
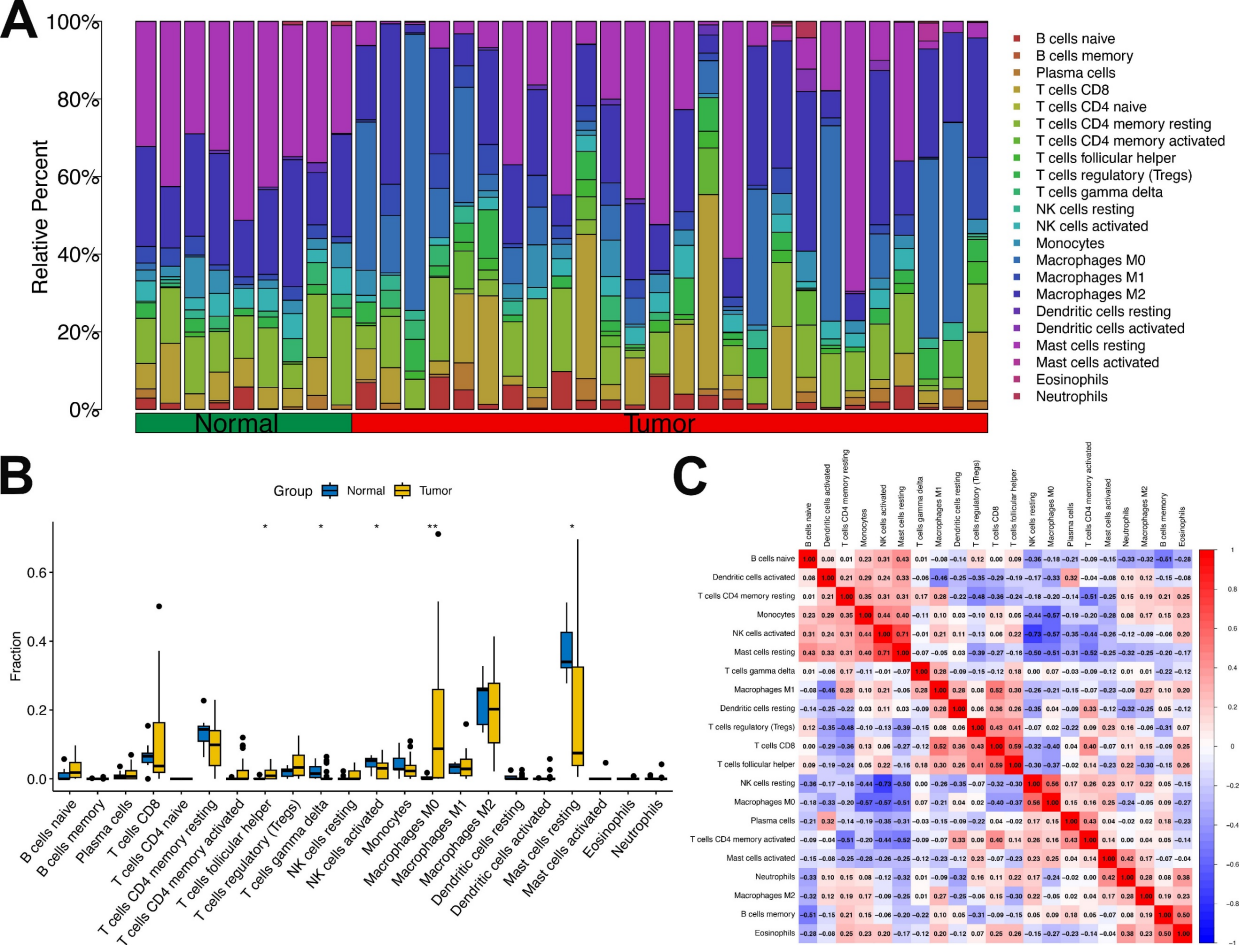
Proportion and correlation analysis of immune cells in the tumor immune microenvironment. **(A)** Proportions of various immune cells in the tumor immune microenvironment between the tumor group and normal group. **(B)** Box plots comparing differences in immune cell proportions between the tumor group and normal group.** (C)** Correlation analysis among various immune cells. *P < 0.05, **P < 0.01.

To delineate the potential cross-talk between Mgenes and the immune regulatory network within the ULMS microenvironment—a vital aspect for deciphering tumor-immune interactions and advancing the development of immunotherapeutic approaches—we first assessed the fractional abundances of various immune cell subsets in the tumor group versus the normal control group (**Figure 10A, B**). Our findings revealed striking disparities in immune cell distribution between the two groups: specifically, follicular helper T cells and M0 macrophages were present at substantially elevated fractional abundances in the tumor group compared to the normal group, a phenomenon that may reflect tumor-driven activation or recruitment of these immune cell populations. In contrast, gamma delta T cells, activated natural killer (NK) cells, and resting mast cells showed notably reduced proportions in the tumor group, which could indicate compromised immune surveillance or a transition toward an immunosuppressive microenvironment. Additionally, the analysis identified heterogeneity in the fractional distribution patterns of immune cells within the tumor group itself, implying that the immune microenvironment may differ across distinct regions or pathological stages of ULMS lesions—variations that could impact the progression of local tumor lesions and responsiveness to treatment.

Furthermore, to delineate the interactive relationships between immune cell populations in the tumor microenvironment, we examined the correlative relationships between distinct immune cell subsets. The results revealed considerable differences in the magnitude and direction of correlations across various immune cell types (**Figure 10C**): for example, some cell subsets displayed positive co-occurrence trends (suggesting potential synergistic interactions), while others exhibited negative associations (indicating reciprocal regulatory inhibition). This observation further underscores the complexity of the immune cell interaction network in the ULMS microenvironment, laying a crucial groundwork for subsequent investigations into the correlations between Mgenes and these immune cell subsets.

**Figure 11:**
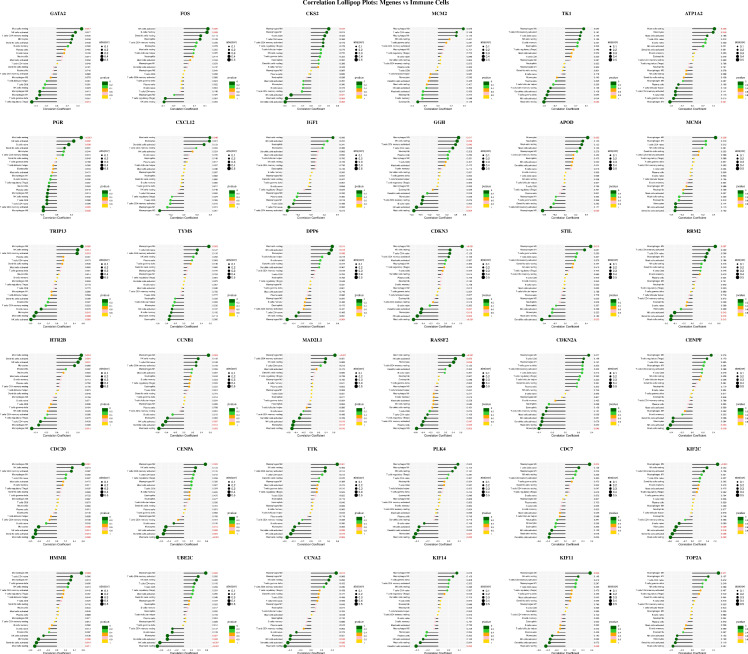
Correlation analysis between Mgenes and various immune cells.

**Figure 12:**
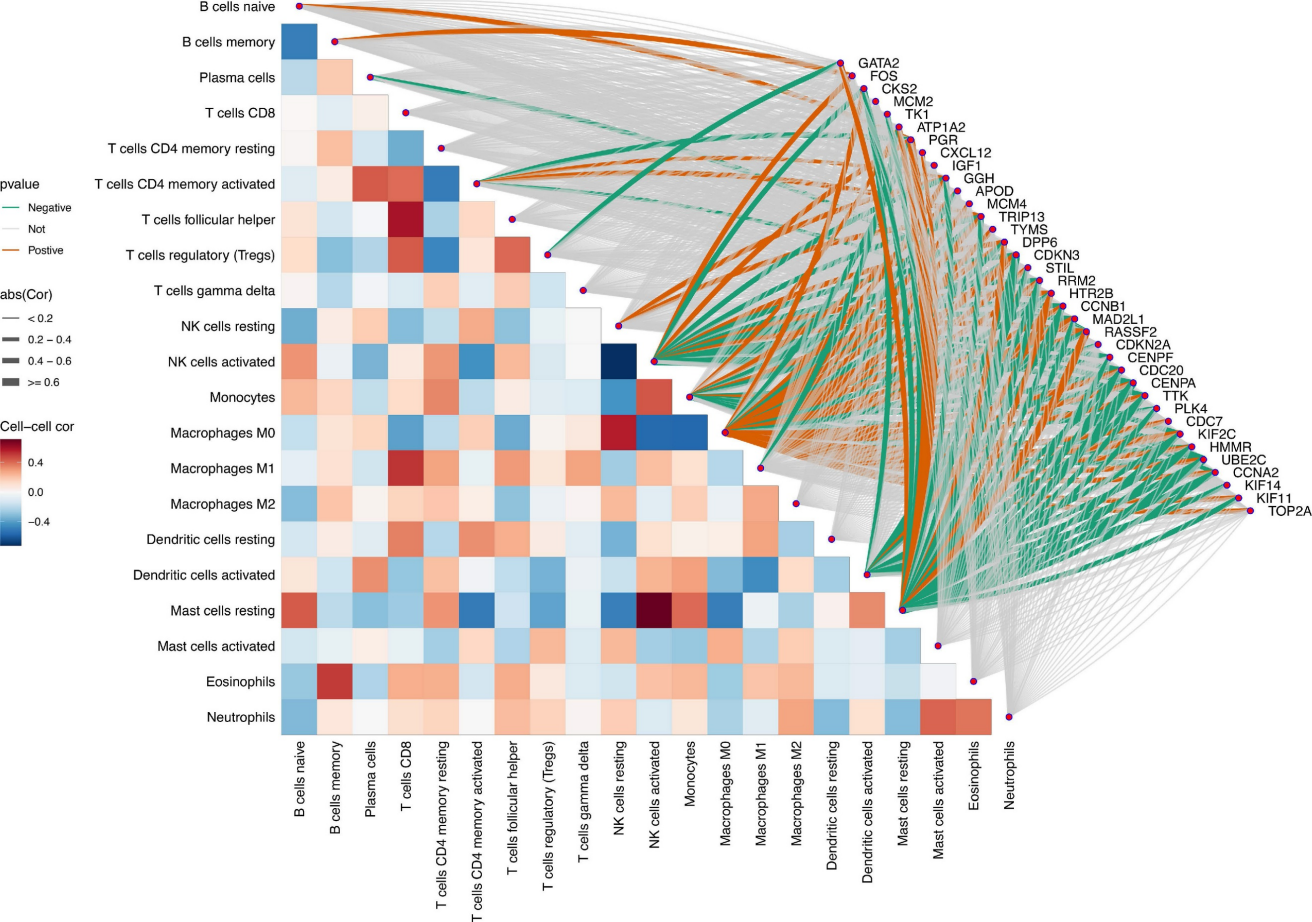
Panoramic correlation heatmap of Mgenes and various immune cells in the tumor immune microenvironment.

After defining the characteristics of immune cell distribution and intercellular correlative relationships, we further analyzed the associations between individual Mgenes and each immune cell subset to elucidate the potential regulatory functions of Mgenes in modulating the tumor immune microenvironment. As illustrated in **Figures 11 and 12**, the correlation patterns differed markedly across distinct Mgenes. Specifically, **Figure 11** presents Spearman correlation lollipop plots depicting the associations between 36 candidate Mgenes and immune cell infiltration patterns in ULMS, with each subpanel dedicated to a single Mgene. The x-axis of each subplot denotes the correlation coefficient, which captures both the direction and magnitude of the relationship between Mgene expression and the cellular abundance of particular immune cell subsets; p-values are indicated adjacent to each data point, with *P* < 0.05 defined as statistically significant—these significant correlations are highlighted in red to differentiate them from non-significant results (marked in black). The key Mgenes analyzed comprised both upregulated and downregulated genes in ULMS, including GATA2, FOS, CKS2, MCM2, TK1, ATP1A2, PGR, CXCL12, IGF1, GGH, APOD, MCM4, TRIP13, TYMS, DPP6, CDKN3, STIL, RRM2, HTR2B, CCNB1, MAD2L1, RASSF2, CDKN2A, CENPF, CDC20, CENPA, TTK, PLK4, CDC7, KIF2C, HMMR, UBE2C, CCNA2, KIF14, KIF11, and TOP2A.

Within the set of statistically significant findings, a cluster of Mgenes that are upregulated in ULMS—including genes associated with cell cycle progression and cellular proliferation (e.g., CKS2, MCM2, TRIP13, TYMS, STIL)—showed positive correlations with immunosuppressive cell subsets, such as M2-like tumor-associated macrophages (TAMs) and regulatory T cells (Tregs). For instance, TRIP13—a gene with high diagnostic relevance in ULMS—exhibited a striking positive correlation with M2 TAMs (*P* < 0.05), suggesting that it may play a role in driving the accumulation of these immunosuppressive cell populations to shape the ULMS tumor immune microenvironment. In contrast, Mgenes that are downregulated in ULMS (e.g., GATA2, CXCL12, HTR2B) displayed significant negative associations with immunosuppressive cells or positive correlations with effector immune cells: GATA2 showed a negative correlation with Tregs and a positive correlation with CD8^+^ cytotoxic T lymphocytes (CTLs), while HTR2B exhibited a negative association with myeloid-derived suppressor cells (MDSCs). Reduced expression of these genes in ULMS may compromise anti-tumor immune responses by disrupting the recruitment of effector cell populations or enhancing the accumulation of suppressive cells.

Taken together, these significant correlations demonstrate that Mgenes are closely linked to the modulation of the ULMS tumor immune microenvironment, with their expression patterns aligning with the characteristic immunosuppressive features of ULMS. Accordingly, the lollipop plots provide visual confirmation that specific Mgenes (both upregulated and downregulated) are statistically significantly associated with specific immune cell subsets—validating their potential utility as biomarkers for ULMS and offering preliminary understanding of their functions in regulating the tumor immune landscape of this disease. Consistent with these findings, **Figure 12** further illustrates the intricate correlations between Mgenes and diverse immune cell populations in the tumor immune microenvironment, indicating that Mgenes play a profound role in regulating the tumor immune microenvironment.

### 3.9 Exploring the correlation between Mgenes and antineoplastic drug sensitivity

**Figure 13:**
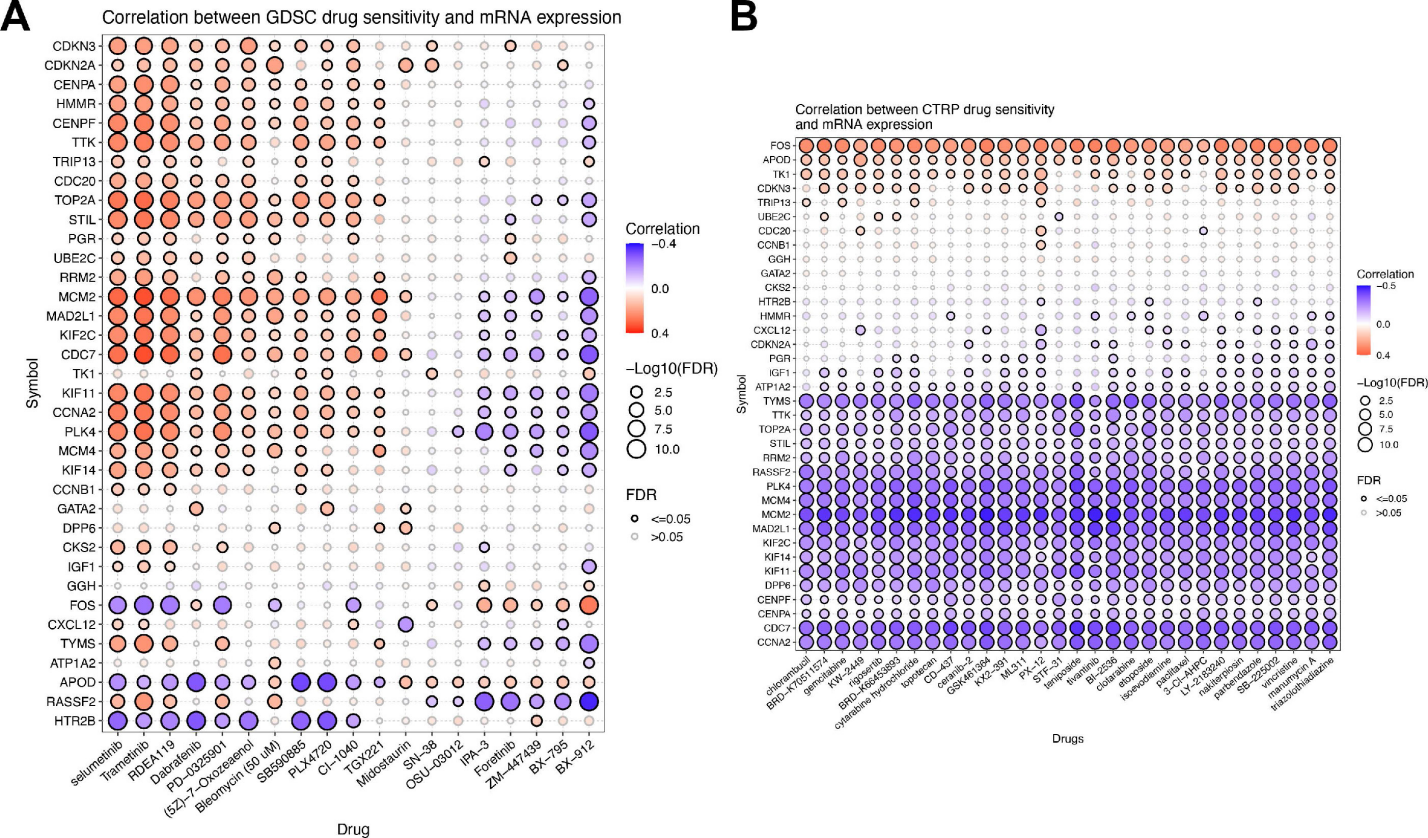
Correlation Analysis of Mgenes Expression and Antineoplastic Drug Sensitivity. (A) Correlation heatmap of antineoplastic drug sensitivity based on the GDSC database, including the top 30 ranked drugs. (B) Correlation heatmap of antineoplastic drug sensitivity based on the CTRP database, including the top 30 ranked drugs. Blue indicates drug sensitivity, and red indicates drug resistance.

To investigate the correlation between Mgenes and antineoplastic drug sensitivity, we performed integrated analyses using the CTRP and GDSC databases. The results derived from the GDSC database are presented in **Figure 13A**, where a correlation heatmap was generated based on the sensitivity profiles of the top 30 ranked antineoplastic drugs. As illustrated in the heatmap, most Mgenes exhibited dual characteristics of both resistance and sensitivity to the panel of drugs dominated by targeted inhibitors. In contrast, the analytical results from the CTRP database are shown in **Figure 13B**, with the heatmap constructed using the sensitivity data of the top 30 antineoplastic drugs. Notably, for the drug panel primarily composed of conventional chemotherapeutic agents, most Mgenes displayed a unimodal pattern of either sensitivity or resistance. Specifically, high expression of FOS, APOD, TK1, and CDKN3 was correlated with antineoplastic drug resistance. In contrast, elevated expression of TYMS, TTK, TOP2A, STIL, RRM2, RASSF2, PLK4, MCM4, MCM2, MAD2L1, KIF2C, KIF14, KIF11, DPP6, CENPF, CENPA, CDC7, and CCNA2 was associated with increased antineoplastic drug sensitivity. Given the prevalent drug resistance in ULMS, the distinct expression patterns of Mgenes may provide valuable insights for the clinical selection of antineoplastic drugs and therapeutic regimens.

### 3.10 Exploring genetically causal links between Mgenes and leiomyosarcoma via mendelian randomization

To explore whether there are genetically causal links between Mgenes and leiomyosarcoma pathogenesis, we performed a Mendelian Randomization (MR) analysis by employing genome-wide association study (GWAS) data and expression quantitative trait locus (eQTL) data. Within this study, eQTL data corresponding to Mgenes were acquired from the IEU Open GWAS Database. Concurrently, a GWAS dataset related to leiomyosarcoma (accession: finngen_R12_C3_LEIOMYOSARCOMA_EXALLC) was sourced from the FinnGen Database, which contained 306 cases and 378,749 controls. Comprehensive information regarding this GWAS dataset—such as sample size, study population, and access URL—is compiled in **Table 1**. Regrettably, the outcomes of our MR analysis failed to provide evidence that supports a genetically causal relationship between Mgenes and leiomyosarcoma susceptibility. This observation implies that genetic variants influencing Mgenes expression may not play a direct causal role in driving leiomyosarcoma development—at minimum within the study populations incorporated here and under the analytical framework adopted in this research.

## 4. Discussion

A critical starting point of this study was the identification of InteGenes—96 genes that overlap between differentially expressed genes (DEGs) in uterine leiomyosarcoma (ULMS) and the WGCNA-derived MEturquoise module, which exhibited the strongest correlation with the ULMS phenotype (correlation coefficient = 0.54, *P* = 6×10⁻⁵). This dual-selection strategy was deliberate: by prioritizing genes that are not only transcriptionally dysregulated between tumor and normal tissues but also central to ULMS-specific co-expression networks, we minimized the risk of focusing on genes with spurious or tissue-unspecific associations, thereby enhancing the biological relevance of our candidate gene set. Functional enrichment analyses further validated this approach, as InteGenes were heavily concentrated in pathways that are well-established drivers of ULMS pathogenesis. Gene Ontology (GO) annotations highlighted processes critical to cell division and genomic stability, including chromosome segregation, nuclear division, and microtubule binding—all of which are frequently dysregulated in ULMS to support uncontrolled proliferation and chromosomal instability. Kyoto Encyclopedia of Genes and Genomes (KEGG) pathway analysis similarly emphasized cell cycle regulation, p53 signaling, and DNA repair mechanisms (e.g., homologous recombination, mismatch repair); for instance, the p53 pathway—often mutated or silenced in ULMS—mediates cell cycle arrest and apoptosis in response to DNA damage, and the enrichment of InteGenes here suggests they may act as downstream effectors of p53 dysfunction, amplifying pro-tumorigenic signaling. Notably, InteGenes also overlapped with hormone-responsive pathways (e.g., progesterone-mediated oocyte maturation), aligning with ULMS's known sensitivity to steroid hormones and the potential for endocrine-based therapeutic strategies. While the relatively small sample size of individual GEO datasets (e.g., GSE68312 with 3 tumor and 3 normal samples) introduced a risk of bias in DEG and module identification, we mitigated this by integrating larger cohorts (TCGA-SARC: 27 ULMS cases; GSE36610: 12 ULMS cases) as the training set and validating findings across three independent datasets, strengthening the robustness of our InteGene prioritization. Future work should further validate these genes in larger, prospectively collected clinical cohorts and explore their functional roles via *in vitro* (e.g., gene knockdown/overexpression in ULMS cell lines) and *in vivo* (xenograft models) experiments to confirm their causal relevance to ULMS progression.

To translate these molecular insights into clinical utility, we compared 113 unique machine learning (ML) algorithms to develop a ULMS diagnostic model—an unprecedented scale of algorithmic comparison for this rare disease. The gradient boosting machine (GBM) model emerged as the top performer, achieving a perfect area under the curve (AUC = 1.0) in the training set and maintaining high accuracy across validation cohorts (GSE764: 92.3%; GSE68295: 100%; GSE68312: 100%). Critically, the model exhibited 100% tumor recall across all datasets, ensuring no ULMS cases were missed—a key attribute for a diagnostic tool, as false negatives could delay treatment initiation and worsen patient prognosis. The 36 core genes of this model (designated “Mgenes”) included both upregulated (e.g., TRIP13, CKS2, MCM2) and downregulated (e.g., GATA2, CXCL12, PGR) transcripts, each with strong individual diagnostic potential (AUC range: 0.782 for HTR2B to 0.972 for TRIP13). TRIP13, which showed the highest individual AUC, is a well-characterized oncogene that regulates mitotic checkpoint control and DNA repair; its overexpression has previously been linked to poor prognosis in other sarcoma subtypes, further validating its relevance to ULMS biology. In contrast, downregulated Mgenes such as GATA2—a transcription factor critical for immune cell development—foreshadowed potential roles in ULMS's immune microenvironment, as discussed below. Compared to prior ULMS diagnostic studies, our model offers distinct advantages: it leverages multi-cohort data to minimize cohort-specific bias, compares a large number of ML algorithms to select the most robust approach (rather than relying on a single method), and captures a broad spectrum of ULMS's molecular signature via both up- and downregulated genes. Clinically, this model could serve as a complementary tool to histopathology, particularly for ambiguous cases (e.g., differentiating ULMS from benign leiomyomas or other uterine sarcomas). However, the model currently relies on transcriptomic data from tissue samples, which requires invasive sampling—a limitation for non-invasive diagnostics. Future research should explore whether Mgene expression can be detected in liquid biopsies (e.g., circulating tumor RNA or exosomes) to develop non-invasive tests, and integrating clinical variables (e.g., patient age, tumor size) with Mgenes may further enhance performance.

The tumor immune microenvironment (TIME) is a key determinant of ULMS progression and response to immunotherapy, yet the molecular regulators linking ULMS's transcriptomic signature to immune dysregulation remain poorly defined. Our CIBERSORT analysis revealed distinct TIME alterations in ULMS: tumor tissues exhibited increased fractions of follicular helper T cells and M0 macrophages (a precursor to immunosuppressive M2-like tumor-associated macrophages [TAMs]) and decreased levels of γδ T cells, activated natural killer (NK) cells, and resting mast cells. These changes align with a well-documented shift toward immune suppression in ULMS: γδ T cells and activated NK cells are critical for innate anti-tumor immunity, and their depletion impairs early tumor surveillance, while M0 macrophage accumulation may facilitate M2 polarization and the secretion of pro-tumorigenic cytokines (e.g., IL-10, TGF-β). Notably, Mgenes exhibited strong correlations with these TIME changes, providing a direct molecular link between ULMS's transcriptome and immune dysfunction. Upregulated Mgenes associated with cell cycle progression (e.g., TRIP13, CKS2, MCM2) showed positive correlations with M2 TAMs and regulatory T cells (Tregs)—two key immunosuppressive populations. For example, TRIP13's positive correlation with M2 TAMs (*P* < 0.05) suggests it may promote M2 polarization, potentially via the secretion of cytokines like CSF1 or activation of STAT3 signaling, which drives macrophage differentiation toward an immunosuppressive phenotype. In contrast, downregulated Mgenes such as GATA2 and CXCL12 correlated negatively with Tregs and positively with CD8^+^ cytotoxic T lymphocytes (CTLs): GATA2 is required for the development and maturation of CTLs and NK cells, so its reduced expression in ULMS may impair effector cell function, while CXCL12—a chemokine that recruits CTLs to tumor sites—may fail to attract anti-tumor immune cells when downregulated. These findings have important implications for immunotherapy: ULMS has shown limited response to immune checkpoint inhibitors (e.g., anti-PD-1), likely due to its highly immunosuppressive TIME. Targeting Mgenes could reverse this suppression—for instance, inhibiting TRIP13 might reduce M2 TAM accumulation, while restoring GATA2 or CXCL12 expression could enhance CTL recruitment. A limitation of this analysis is that CIBERSORT infers immune cell fractions from bulk RNA-seq data, which cannot capture single-cell-level heterogeneity (e.g., subpopulations of M2 TAMs with distinct functional roles). Single-cell RNA sequencing of primary ULMS tissues would further refine our understanding of Mgene-TIME interactions and identify more precise immune targets.

To address whether Mgenes exert causal genetic effects on ULMS susceptibility—a key question for distinguishing driver vs. passenger genes—we performed Mendelian randomization (MR) analysis using expression quantitative trait locus (eQTL) data from the IEU Open GWAS Database and a ULMS genome-wide association study (GWAS) dataset from FinnGen (306 cases, 378,749 controls). Surprisingly, no evidence of a genetic causal relationship was found, suggesting that genetic variants influencing Mgene expression do not directly drive ULMS development in the studied populations. Several factors may explain this null result. First, the FinnGen ULMS dataset includes only 306 cases, which limits statistical power to detect modest causal effects—particularly for rare genetic variants, given ULMS's low incidence. Large-scale GWAS for ULMS are challenging due to its rarity, and future studies should integrate data from multiple cohorts (e.g., UK Biobank, expanded TCGA-SARC) to increase sample size and power. Second, the eQTL data used in MR may not reflect Mgene expression in uterine tissues: most public eQTL datasets are derived from blood or non-uterine organs, and tissue-specific eQTLs could be missed, leading to inaccurate estimates of SNP-Mgene associations. Third, Mgenes may be consequences rather than causes of ULMS: their differential expression could result from epigenetic modifications (e.g., DNA methylation, histone acetylation) or cues from the tumor microenvironment (e.g., cytokine signaling) rather than genetic variation. Finally, ULMS exhibits high genetic heterogeneity, with distinct subtypes driven by different driver mutations (e.g., TP53, ATRX); our MR analysis did not stratify by subtype, which may have masked subtype-specific causal effects. Despite being negative, this result is valuable: it rules out Mgenes as major genetic drivers of ULMS, guiding future research toward non-genetic mechanisms underlying their dysregulation (e.g., epigenetic or post-translational modification). Additionally, it prevents overinterpretation of Mgenes as genetic susceptibility markers, which could misdirect clinical screening efforts. Future MR studies should use larger, tissue-matched eQTL and GWAS datasets to confirm these findings.

Collectively, these discussions highlight the multi-faceted contributions of our study: from identifying biologically relevant candidate genes (InteGenes) to developing a clinically useful diagnostic model (based on Mgenes), uncovering links between Mgenes and ULMS's immunosuppressive TIME, and clarifying the non-genetic role of Mgenes in ULMS pathogenesis. Each finding addresses a critical gap in current ULMS research, while acknowledging limitations that point to future directions—ultimately advancing our understanding of this aggressive disease and laying the groundwork for improved diagnostics and therapies.

## 5. Conclusion

This study systematically investigated the molecular landscape of uterine leiomyosarcoma (ULMS) using multi-cohort transcriptomic data, weighted gene co-expression network analysis (WGCNA), machine learning, tumor immune microenvironment (TIME) analysis, and Mendelian randomization (MR). Key findings include: (1) Identification of 96 InteGenes enriched in core oncogenic pathways (cell cycle, p53 signaling, DNA repair), which are central regulators of ULMS pathogenesis; (2) Development of a gradient boosting machine (GBM)-based diagnostic model (36 Mgenes) with excellent performance (training AUC = 1.0, validation accuracy 92.3-100%); (3) Mgenes are closely associated with ULMS's immunosuppressive TIME, with upregulated Mgenes correlating with M2 tumor-associated macrophages (TAMs)/regulatory T cells (Tregs) and downregulated ones with effector immune cells; (4) Preliminary correlation between Mgenes expression profiles and antineoplastic drug sensitivity/resistance; (5) No genetic causality between Mgenes and ULMS via MR, indicating potential non-genetic mechanisms in Mgene dysregulation.

These findings provide novel insights into ULMS's molecular and immune biology, and identify Mgenes as promising diagnostic biomarkers and immunotherapeutic targets, laying a solid foundation for translational research. Clinically, although the GBM model relies on postoperative samples (inherent to current ULMS diagnosis), it serves as a valuable auxiliary tool for pathological confirmation—especially in ambiguous cases—helping reduce misdiagnosis of this rare malignancy. More importantly, Mgenes' correlations with TIME components (e.g., M2 TAMs, Tregs) and drug sensitivity open new avenues for personalized treatment: Mgenes highly expressed with M2 TAMs/Tregs may be targets for immunotherapies (e.g., TAM repolarization agents, checkpoint inhibitors), while those associated with drug sensitivity/resistance can guide neoadjuvant or adjuvant therapy selection, addressing the unmet clinical need for precise ULMS management.

Notable strengths of this study include integration of multi-cohort data (GEO, TCGA-SARC, single-cell sequencing) and a comprehensive analytical framework, ensuring robust and generalizable findings; additionally, focusing on Mgenes advances understanding of ULMS's immunosuppressive microenvironment, a key treatment barrier. However, limitations exist: (1) Findings are based on retrospective transcriptomic data, requiring prospective validation in larger, diverse cohorts; (2) The functional mechanisms of Mgenes in regulating TIME and drug sensitivity remain unclear, needing in-depth *in vitro*/*in vivo* verification; (3) Non-genetic mechanisms (e.g., epigenetic modification) suggested by MR results require further exploration.

Future research will focus on prospective validation of Mgenes, elucidating their functional roles in ULMS progression and immune regulation, and testing Mgene modulation to enhance immunotherapy/chemotherapy efficacy in preclinical models. Ultimately, this work aims to improve ULMS's precise diagnosis, prognostic stratification, and personalized treatment, thereby enhancing outcomes for patients with this highly aggressive malignancy.

## Supplementary Material

Supplementary code, figures and tables.

## Figures and Tables

**Table 1:** Characteristics of GWAS dataset included in the study

ID	Description	Cases (n)	Controls (n)	Population	URL
finngen_R12_C3_LEIOMYOSARCOMA_EXALLC	Leiomyosarcoma, excluding all cancers (controls excluding all cancers)	306	378749	European	https://storage.googleapis.com/finngen-public-data-r12/summary_stats/release/finngen_R12_C3_LEIOMYOSARCOMA_EXALLC.gz

## Data Availability

All datasets utilized and/or analyzed in the present study are publicly accessible. Specifically, four transcriptomic datasets were obtained from the Gene Expression Omnibus (GEO) database (https://www.ncbi.nlm.nih.gov/geo/) under the following accession numbers: GSE36610, GSE764, GSE68312, and GSE68295. Additionally, the TCGA-SARC dataset (relevant to uterine leiomyosarcoma) was retrieved from The Cancer Genome Atlas (TCGA) database (https://portal.gdc.cancer.gov/), which provided RNA-seq data and corresponding clinical information for the study.
