# Supplementary figures and images for: Identification of Key Genes via Integrated Multi-Omics and Machine Learning Uncovers Tumor Biological Features and Prognostic Biomarkers in Uterine Leiomyosarcoma
Source: Int J Med Sci. 2026 Feb 4;23(3):927–49. doi: 10.7150/ijms.126491 (PMC12964573; doi:10.7150/ijms.126491)

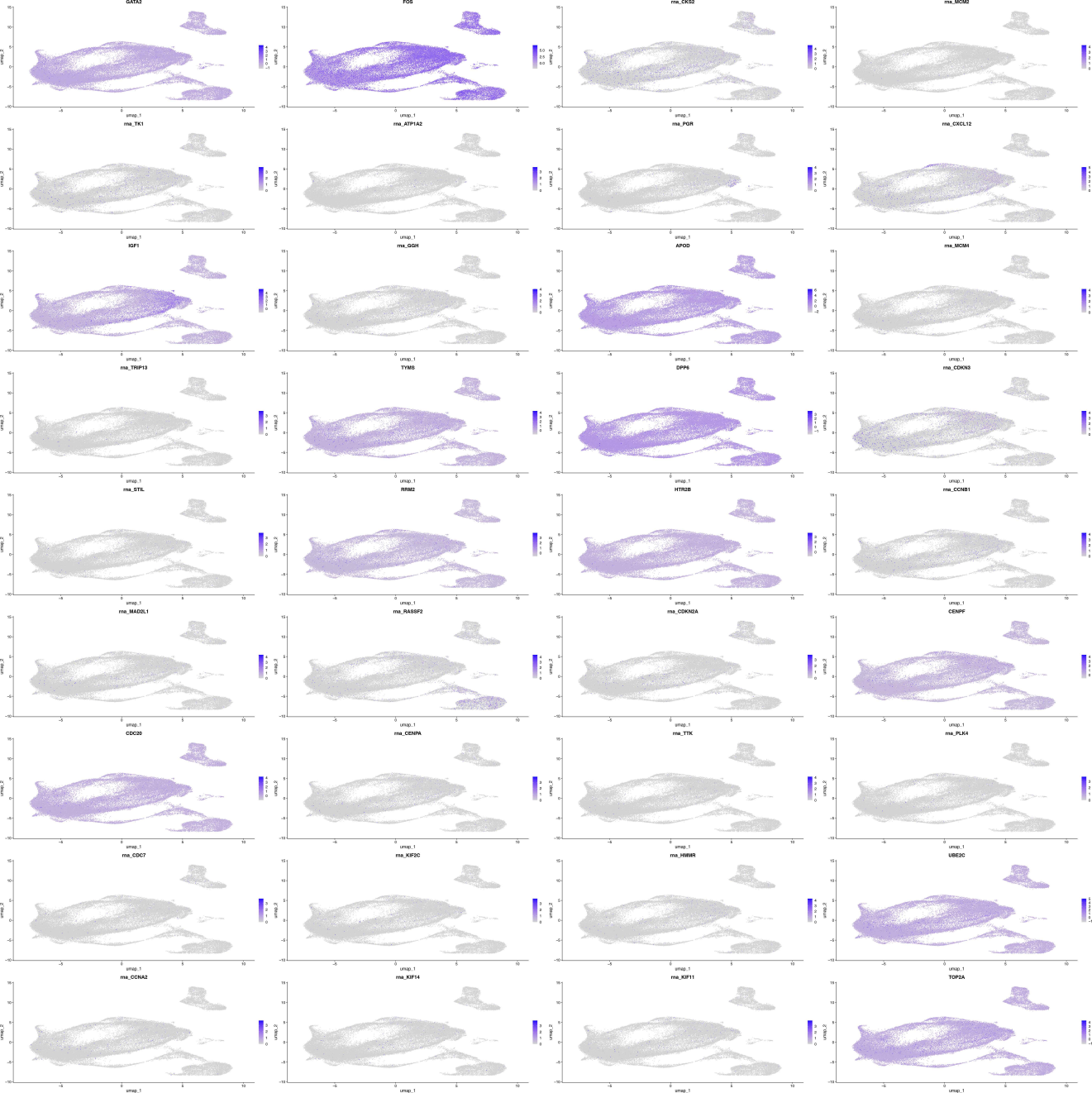

Supplement: Supplementary file 1 — Supplementary code, figures and tables. [file ijmsv23p0927s1.zip › Supplementary Figure 1.pdf]

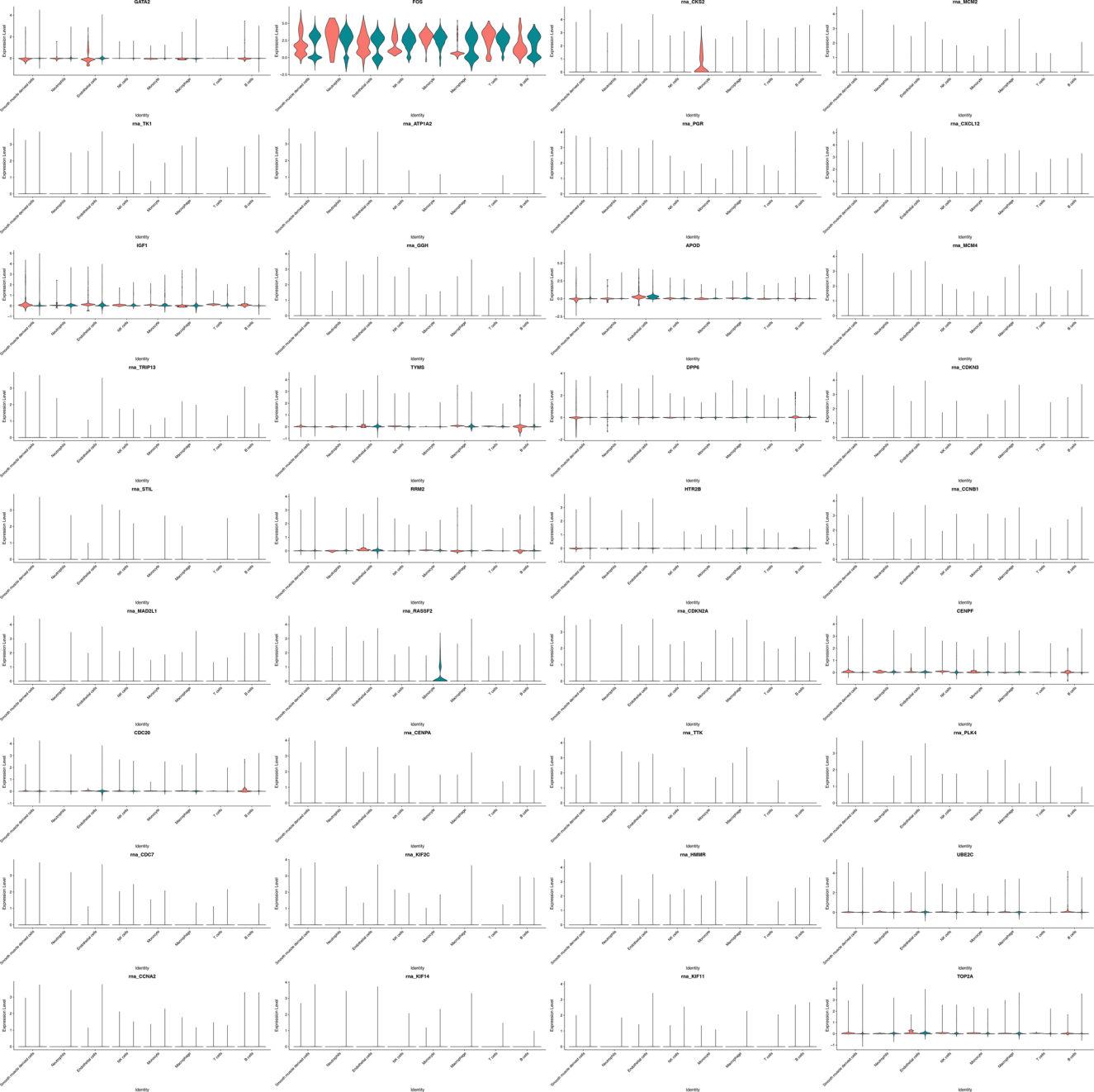

Supplement: Supplementary file 1 — Supplementary code, figures and tables. [file ijmsv23p0927s1.zip › Supplementary Figure 2.pdf]
